# Social Perceptions and Attitudes Towards Free-Roaming Cats and Dogs in Portugal: An Exploratory Study

**DOI:** 10.3390/ani15060771

**Published:** 2025-03-08

**Authors:** Alexandre Azevedo, Filipa Peste, Paloma Linck, João Carvalho, Danielle Crawshaw, Eduardo Ferreira, Rita Tinoco Torres, Victor Bandeira

**Affiliations:** 1 CIVG—Vasco da Gama Research Center, EUVG—Vasco da Gama University School, 3020-210 Coimbra, Portugal; 2 Departamento de Biologia, Universidade de Aveiro, Campus Universitário de Santiago, 3810-193 Aveiro, Portugal; 3 CESAM & Departamento de Biologia, Universidade de Aveiro, Campus Universitário de Santiago, 3810-193 Aveiro, Portugal

**Keywords:** animal welfare, pet ownership, social attitudes, social perceptions, biodiversity, public health

## Abstract

Free-roaming cats and dogs impact biodiversity, public health, and the welfare of other animals. Attitudes towards free-roaming animals can influence their population dynamics and management success. We conducted an online survey to evaluate social perceptions and attitudes towards free-roaming animals among self-selected Portuguese residents aged 18 or older with internet access. The survey focused on responsible ownership, perceptions and attitudes, and management practices, and allowed the collection and analysis of 1083 responses (607 for dogs and 476 for cats). Our results identified needs for improvement in pet ownership: increasing pet cat identification, reducing unsupervised outdoor access, and promoting pet dog sterilization. In terms of management strategies, we found strong support for trap–neuter–release, sheltering, sanctions on abandonment, and educational campaigns. We also found limited support for lethal control methods and fear of culling and long-term caging as barriers to reporting free-roaming animals. While our findings are based on a self-selected online sample, they establish a foundation for future research while also offering valuable guidance for policymakers and stakeholders.

## Simple Summary

Free-roaming cats and dogs pose significant challenges to biodiversity, public health, and animal welfare. We surveyed 1083 people in Portugal to understand public perceptions and attitudes towards these animals. Our findings highlight areas for improvement in pet ownership, including increasing identification and registration of cats, reducing their unsupervised outdoor access, and promoting sterilization for dogs. Respondents showed a strong concern for the welfare of free-roaming animals, supporting methods like trap–neuter–release for cats, sheltering for dogs, sanctions on abandonment, and educational campaigns. However, there was little support for lethal control methods, and fear of culling or long-term confinement was a major barrier to reporting free-roaming animals. These insights can help policy makers and researchers develop socially accepted strategies that balance animal welfare with effective population management.

## 1. Introduction

With global population estimate exceeding half a billion each [1,2], dogs and cats are the most predominant carnivores on the planet, with a total biomass exceeding all terrestrial wild mammals combined [3]. Free-roaming cats and dogs are defined as owned or unowned animals that have unsupervised access to outdoor environments [4,5]. Their ability to roam and breed freely in the natural environment can lead to several negative impacts on the animals themselves, but also on other domestic animals [6], wildlife [7,8], and on public health [9,10], which has led to efforts around the globe to manage these free-roaming populations [11].

Free-roaming animals are at risk for vehicle collisions, animal attacks, poisoning, disease, and persecution (reviewed in [12]), all of which are associated with significant suffering and welfare impacts with durations that can vary from a few seconds or minutes (e.g., vehicle collisions) to several months (e.g., chronic disease). Free-roaming cats have been shown to negatively impact wildlife through predation [7,13,14], disease transmission [15,16], hybridization [17], and to hold potential for zoonotic disease transmission [10,18]. Free-roaming dogs have also been shown to impact wildlife through disease and predation [8,19], and are associated with an array of public health impacts that range from bites [20] to the transmission of rabies to humans over the last 4000 years [21]. While the role of free-roaming dogs in rabies transmission is acknowledged globally [9], free-roaming dogs and cats are capable of transmitting many other zoonoses whose impacts are often underestimated [22,23,24]. Examples of zoonoses that can be transmitted by cats and dogs include cat scratch disease (*Bartonella henselae*), leishmaniasis (*Leishmania infantum*), tuberculosis, echinococcosis (*Echinococcus multilocularis*), toxoplasmosis (*Toxoplasma gondii*), and toxocariosis (*Toxocara canis* and *Toxocara cati*), but many diseases are of concern due to unconfirmed but potential transmission from pets such as avian influenza (H5N1), noroviruses, and rotaviruses [22,23,24].

Although there are no estimates regarding the dimension of free-roaming cat and dog populations in Portugal, government shelters collected 184,000 free-roaming cats and dogs between 2018 and 2022, averaging 37,000 animals per year [25]. Nevertheless, there are some studies highlighting the impacts of free-roaming dogs and cats, such as reports of hybridization with the Iberian wolf (*Canis lupus signatus*) [26] and the European wildcat (*Felis silvestris silvestris*) [27]; predation on livestock and several species of wildlife [6], including endangered island seabird colonies [28,29]; and the potential for zoonosis transmission [10,30,31,32,33,34].

To prevent or mitigate these impacts, it is necessary to understand the factors that influence the free-roaming dog and cat populations. While a variety of management approaches exist—including several levels of confinement, trap–neuter–release, and community caretaking—their effectiveness depends on local contexts and public attitudes [35]. Human behavior and attitudes towards free-roaming animals can influence their population dynamics and the success of management actions. In the context of stray dog population control, the World Organisation for Animal Health defines carrying capacity as “the upper limit of the dog population density that could be supported by the habitat” and lists human acceptance, attitudes, and behavior as determinants of carrying capacity alongside food, water, and shelter [36]. Additionally, pet ownership behaviors influence the number of cats or dogs that are sterilized, allowed to roam freely, or abandoned, therefore influencing the contribution of pets as sources for free-roaming cat and dog populations [36,37]. Finally, human attitudes and perceptions influence the acceptance, feasibility, and success of preventative and management measures [35,38,39,40]. Therefore, knowledge of the human behaviors related to pet ownership and social attitudes toward free-roaming animals and their management is essential to design and implement successful management efforts.

Studies focusing on social attitudes towards free-roaming cats and dogs are increasing in the literature [4,5,11,39,41,42,43], highlighting the importance of this topic in the context of population management and control. Portugal is no exception and survey studies have been undertaken focusing on pet ownership practices as key determinants of the welfare of cats and dogs [44] and zoonotic risk [45]. However, current discussions of a national strategy to manage free-roaming animals and their impacts [46] require a broader assessment of social attitudes and perceptions. To address this gap, this study aims to assess social attitudes that may influence the occurrence of free-roaming dog or cat populations and their management at a country-wide scale. We performed a cross-sectional exploratory survey study focusing on ownership practices, perceptions and social attitudes in Portuguese residents aged 18 or older with internet access. This study is essential to inform current and future strategies to successfully manage free-roaming dog and cat populations and their impacts in Portugal in a way that safeguards animal welfare and is aligned with predominant social norms in the country.

## 2. Materials and Methods

The study used online questionnaires targeting people over 18, living in Portugal. Due to the differences in social attitudes towards cats and dogs, an independent questionnaire was designed for each species. The questionnaires were accessible between 20 May 2023 and 10 July 2023 through the official websites of the Institute for the Conservation of Nature and Forests (ICNF) and of the University of Aveiro. To broaden the reach and minimize sampling bias from overrepresentation of people related to the research field, the survey links were disseminated via the social media platform Facebook (www.facebook.com). A targeted paid campaign was implemented using Facebook Ads Manager, which aimed to enhance user interaction by increasing clicks on relevant links. The advertisement was promoted exclusively on Facebook for a duration of seven days, targeting users aged 18 and older residing in Portugal. The campaign objective, as defined within Facebook’s advertising framework, was to maximize interactions with the post. As participation in online surveys is voluntary, representativeness can be affected by biases from self-selection and access to digital media.

This study was performed in compliance with the norms of the ethical committee of the University of Aveiro. It was conducted in Portugal, collecting exclusively anonymous data from participants. In accordance with Regulation (EU) 2016/679 of the European Parliament and of the Council of 27 April 2016 on the protection of natural persons with regard to the processing of personal data and on the free movement of such data, Official Journal of the European Union, L119, 4 May 2016, pp. 1–88, and Portuguese Law No. 58/2019, which implement data protection principles, the collection of fully anonymized data does not necessitate specific ethical approval. Therefore, ethical approval was not sought for this study. All participants provided informed consent following comprehensive disclosure of the study’s objectives and procedures. At the start of the questionnaire, participants were informed of the purpose of the study, provided links to the project websites and an email contact for the research team, and informed that participation was voluntary and could be withdrawn at any point before submission and that all data collected were anonymous. They were then asked to provide their consent by choosing between “yes” or “no” in response to whether they agreed that they had (i) read and understood the explanation of the study, (ii) understood the purpose of the study and were aware of the contacts for any questions, (iii) were aware that they could abandon the survey at any time before submission, and (iv) agreed to the storage and utilization of the anonymous data for the purpose of the study.

Questionnaires were designed in Portuguese. The first section contained closed questions on the ownership practices of the respondent, regarding the targeted species. The second section contained closed questions and questions on a five-point Likert scale focusing on respondents’ attitudes toward free-roaming animals and options for their management. The final section contained questions regarding the sociodemographic information of the respondent. The language and format of the questions were kept as simple and objective as possible. An initial version of the questionnaires was piloted by the authors to identify errors, inconsistencies, and potential ambiguities. Based on this internal review, revisions were made to improve clarity and coherence. The reviewed version was then piloted and reviewed by an external researcher, experienced in the study of human-animal relations and social science methodologies. Feedback from this second pilot led to further refinements, including rewording unclear questions and refining response formats and categories. No substantive changes were made to the core content of the questionnaire. The final versions of the questionnaires are available in the Supplementary Information (File S1). Two questions were used to assess attitudes toward free-roaming dogs and using ordinal regression models. The first asked participants to indicate their agreement with the statement: “I like the presence of free-roaming dogs around my house or workplace,” with responses provided on a five-point Likert scale (strongly disagree, disagree, neither agree nor disagree, agree, strongly agree), along with a non-response option. The second question assessed preferences for the presence of free-roaming dogs based on the question “What would you prefer?” with the following response options: I prefer there to be no free-roaming dogs, I prefer there to be fewer free-roaming dogs, I do not mind the presence of free-roaming dogs, I prefer there to be more free-roaming dogs, along with a non-response option. These variables were used in statistical models to explore factors influencing tolerance toward free-roaming dogs and public preferences for their management.

Complete submissions were extracted and considered for analysis. Dog and cat questionnaires were analyzed separately. Visualization of Likert scale data on the attitudes towards free-roaming animals was performed using horizontal bar plots and the potential for conflict index data visualization method [47,48,49]. The potential for conflict index is a standardized approach used to assess consensus and disagreement in Likert scale responses. It is calculated using the mean to represent the central tendency of the responses and the variance, normalized to a scale from zero to one, to reflect a range from complete agreement (variance is zero) to maximum disagreement (variance is one). The potential for conflict index is represented through bubble plots, where the location of the bubbles on the vertical scale indicates the mean of the answers and the diameter of the bubble indicates the normalized variance in the answers [49]. For descriptive analyses, levels of five-point Likert scales were simplified by grouping levels totally disagree with disagree, and totally agree with agree, to obtain a three-level scale of disagree, neutral, agree.

Analyses to determine which factors influence responsible ownership practices and attitudes towards free-roaming animals were performed using R software version 4.0.3 [50] using ordinal regression modeling with the ordinal package [51]. To assess the influence of relevant factors on reported responsible pet-ownership practices, we calculated an index of responsible ownership (Table 1) based on the sum of six indicators included in the questionnaires on a scale from zero (least responsible ownership) to six (most responsible ownership). For each indicator [identification and registration, allowing to breed, sterilization, allowing to roam free, “first level” vaccination (rabies for dogs, RCP for cats), “second level vaccination” (other vaccines for dogs, FeLV vaccine for cats)], a score of one was assigned to the responsible option and a score of zero was assigned to the least responsible option (e.g., reporting rabies vaccination for dogs would be scored one, while reporting not to would be scored zero). Cases with responsible ownership index scores between zero and two were collapsed into a single level due to the limited number of observations.

Ordinal regression modeling was performed in three steps: (a) ordinal regression models were built with candidate factors based on their biological relevance and prior literature; (b) stepwise model selection based on a likelihood ratio test was performed using the drop1 function, where variables were retained if they improved model fit (as indicated by statistically significance (*p* < 0.05) in likelihood ratio tests (LRT)); (c) model assumptions were verified using the generalhoslem [52] and brant [53] packages. Separate models were built for dogs and cats. Models to assess the influence of candidate factors on attitudes towards the existence and presence of free-roaming animals followed the same procedure. Dependent and independent variables used in inferential analyses are presented in Table 2. Results for the final models are presented as odds ratios, with *p*-values (α = 0.05) and 95% confidence intervals (OR, *p*-value, 95% CI). Separate models were built for answers to dog and cat questionnaires. For the final ordinal regression models, post-hoc power analysis was conducted using Cohen’s f^2^ derived from Nagelkerke’s adjusted R^2^, computed via the rcompanion package [54] and analyzed using the pwr package [55]. The responsible ownership models (model 1) for cats and dogs had estimated powers of 99.98% (f^2^ = 0.11, α = 0.05) and 99.97% (f^2^ = 0.094, α = 0.05), respectively. The models focusing on tolerance to free-roaming animals (model 2) had an estimated power of 99.72% (f^2^ = 0.057, α = 0.05) for dogs and 85.65% (f^2^ = 0.022, α = 0.05) for cats. The models focusing on the acceptability of free-roaming animals (model 3) had an estimated power of 95.03% (f^2^ = 0.025, α = 0.05) for dogs and 99.29% (f^2^ = 0.053, α = 0.05) for cats. These values indicate that all models had the ability to detect small-to-moderate effect sizes within the surveyed sample.

## 3. Results

We received 607 and 476 valid responses for dogs and cats, respectively. At the date of data extraction for analysis (10 July 2023), the social media platform service estimated having reached 60,832 users, resulting in an engagement rate of 5.91%. Respondents accessed the questionnaire primarily through three sources: the University of Aveiro (UA) website, Facebook, and the ICNF website. For the dog survey (*n* = 607), the most frequent sources were UA (39%), Facebook (30%), and ICNF (16%), with a smaller number of responses originating from Instagram (3%) and other sources (1%). Similarly, for the cat survey (*n* = 476), the main sources were UA (44%), Facebook (34%), and ICNF (17%), while Instagram (4%) and other sources (1%) contributed fewer responses.

Among the 607 respondents to the dog survey, 436 (72.0%; 95% CI: 68.4–75.2%) were dog owners, and among the 476 respondents to the cat survey, 373 (78.4%; 95% CI: 74.6–81.9%) were cat owners. Submissions were biased toward female respondents, with 79.0% (95% CI: 75.8–81.8%) and 81.3% (95% CI: 77.5–84.7%) female respondents for dogs and cats, respectively. A varied distribution across all age classes was obtained, as well as answers from all 18 districts of Portugal, with Lisbon, Aveiro, Porto, and Setúbal being the most represented. The social and demographic distribution of the respondents is provided in the Supplementary Information (Table S1).

### 3.1. Responsible Ownership Practices

**Figure 1:**
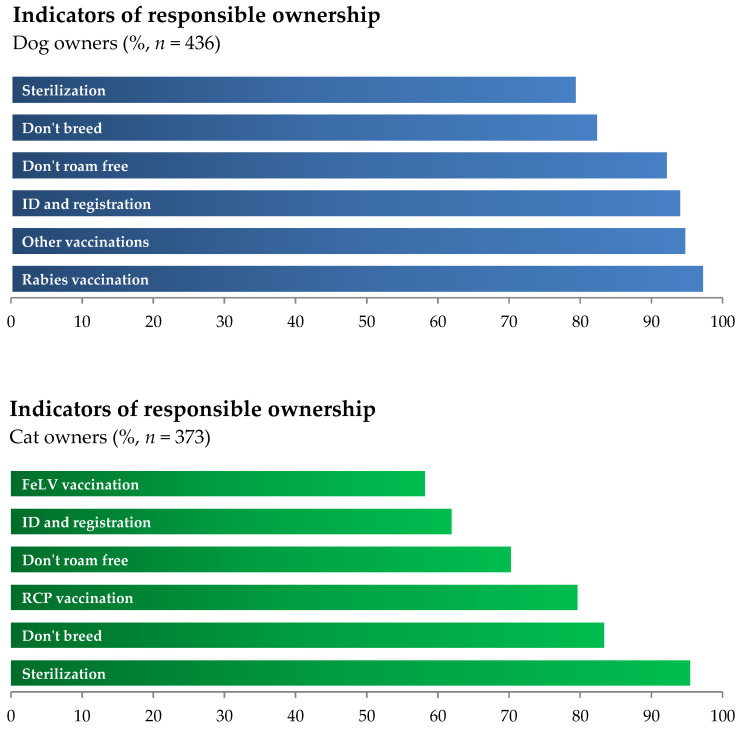
Responsible ownership indicators for dogs (blue, *n* = 436) and cats (green, *n* = 373) based on self-reported data from questionnaires (relative frequencies). Legend: Sterilization—owners who report surgically sterilizing their dogs/cats; Don’t breed—owners who report they never allow their dog/cats to breed; Don’t roam—owners who report they never allow their dogs/cats outdoor access unaccompanied; ID and registration—owners who report they identify and register all their dogs/cats; Other vaccinations—owners who report vaccinating their dogs with vaccines other than mandatory rabies vaccines; Rabies vaccination—owners who report vaccinating their dogs against rabies; FeLV vaccination—owners who report vaccinating their cats against feline leukemia virus; RCP vaccination—owners who report vaccinating their cats with a trivalent vaccine.

Regarding responsible dog ownership indicators (Figure 1), 80% (95% CI: 76.0–83.0%) of the surveyed owners reported compliance with all indicators. Rabies vaccination (97.5%; 95% CI: 96.0–99.0%), animal adoption (95.2%; 95% CI: 93.0–97.0%), vaccination against diseases other than rabies (95%; 95% CI: 92.0–97.0%), and animal identification and registration in the Companion Animal Information System (SIAC—Sistema de Informação de Animais de Companhia) (94.3%; 95% CI: 91.0–97.0%) were virtually ubiquitous. Eighty percent (95% CI: 76.0–83.0%) of the dog owners reported using surgical sterilization as a form of contraception, 83% (95% CI: 79.0–86.0%) reported never having a litter from a dog they owned, and 92% (95% CI: 89.0–94.0%) reported never allowing their dogs to have unsupervised access to the street. In the case of cats, approximately 60% (95% CI: 55.0–65.0%) of the surveyed owners reported compliance with all indicators. Surgical sterilization (95.4%; 95% CI: 93.0–97.0%) was the indicator with the highest compliance rate among cats, and 83.4% (95% CI: 80.0–87.0%) of owners reported never having reproduction among their cats. Vaccination was provided by 79.6% (95% CI: 75.0–83.0%) of owners, of which 58.2% (95% CI: 53.0–64.0%) vaccinated their cats against feline leukemia. Around 70% (95% CI: 65.0–75.0%) of owners reported never allowing their cats unsupervised access to the street, and 61.9% (95% CI: 57.0–67.0%) identify and register all their cats. The cost of surgical contraception was the barrier most frequently reported by owners (46%; 95% CI: 41.0–51.0% for dog owners and 50%; 95% CI: 45.0–55.0% for cat owners) and was the only barrier mentioned by more than 5% of the respondents.

**Figure 2:**
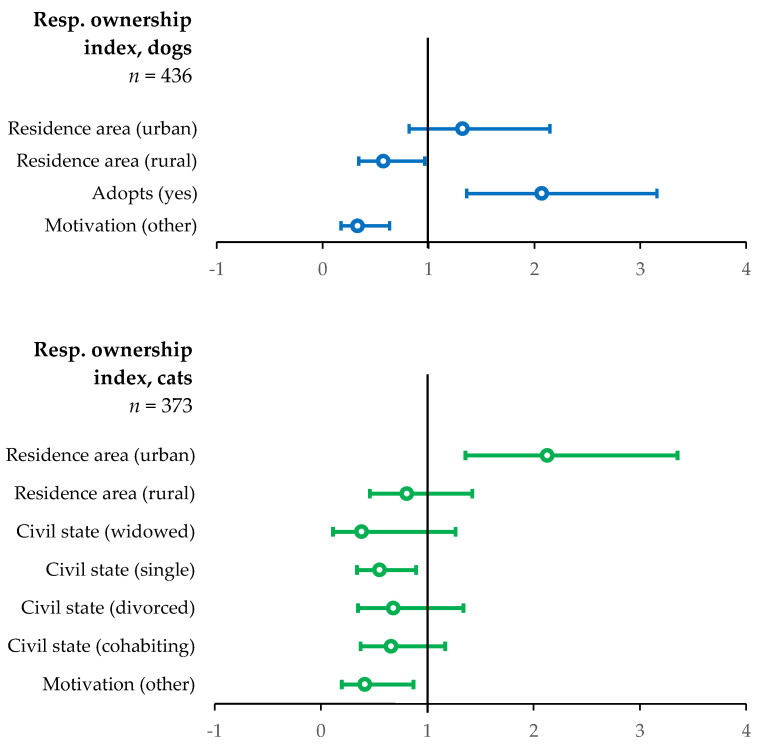
Factors that influence responsible ownership index. Odds ratios (dots) and 95% confidence intervals (whisker bars) of the final model for the variation of the responsible ownership index in dog (blue, *n* = 436) and cat (green, *n* = 373) owners.

In the ordinal regression model assessing the factors that may influence responsible ownership index scores for dog owners (*n* = 390), the factors retained were motivation for ownership, adoption practice, and typology of residential area (Table 3, Figure 2). Owners who chose motivations other than companionship for owning dogs were associated with lower scores of responsible ownership index than those who chose companionship (OR = 0.33; 95% CI = 0.17–0.63; *p* = 0.001). Owners in the sample who reported adopting dogs as a means of acquisition were associated with higher levels of responsible ownership index (OR = 2.07, 95% CI = 1.36–3.16; *p* = 0.001) than those that reported not adopting. Finally, owners residing in rural or natural areas were associated with a lower score of responsible ownership index (OR = 0.57; 95% CI = 0.34–0.96; *p* = 0.036) than residents of peri-urban areas.

In the sample of cat owners (*n* = 334), the factors associated with differences in the responsible ownership index that were retained in the final model were motivation for ownership, marital status, and typology of residential area (Figure 2). Owners who cited motivations for owning cats other than companionship were associated with lower responsible ownership scores than those who cited companionship as the reason for owning cats (OR = 0.41; 95% CI = 0.20–0.87; *p* = 0.02). Compared to married owners in the sample, single owners (OR = 0.55; 95% CI = 0.34–0.90; *p* = 0.016) were associated with lower responsible ownership index scores. Finally, owners residing in urban areas were associated with higher levels of responsible ownership index (OR = 2.13; 95% CI = 1.36–3.35; *p* = 0.001) compared to residents of peri-urban areas.

### 3.2. Attitudes Towards Free-Roaming Dogs

**Figure 3:**
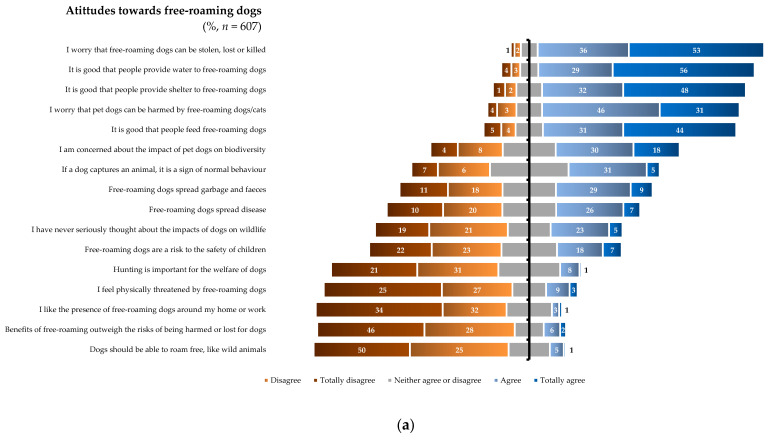

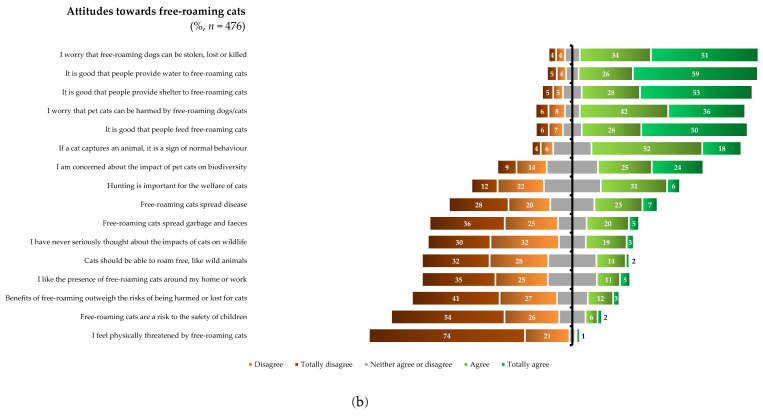
(**a**). Attitudes toward free-roaming dogs based on questionnaire answers. (**b**). Attitudes toward free-roaming cats based on questionnaire answers.

Among the 607 respondents, 70.5% reported having at some point provided food to free-roaming dogs, 65.2% had provided water, 37.1% shelter, and 17.1% had provided other care. Most respondents agreed that people should provide food, water, or shelter to free-roaming dogs (Figure 3a). Respondents’ opinions on hygiene and public health risks varied, with 32.9% agreeing that free-roaming dogs spread diseases and 37.9% agreeing that they spread litter and feces. Most of the respondents expressed concern about the welfare impacts on the free-roaming dogs themselves (88.0%) or on companion animals (77.4%), but only 48.6% agreed upon the impacts of dogs on wildlife, and 28.2% of respondents stated they had never seriously thought about it. Opinions varied markedly on whether predation is a sign of normal behavior in dogs (35.7% agreed, 30.8% neutral, 30.8% disagreed), but most respondents disagreed that hunting animals is necessary for the welfare of dogs (65.6%). Over a quarter of the respondents (27.2%) reported having felt physically threatened by a free-roaming dog at some point in their life, of which 7.2% had been attacked, and 1.6% had been bitten or had seen a family member bitten in the year preceding the survey. Only a small proportion of the respondents reported currently feeling physically threatened by free-roaming dogs (12%), but when it came to children, 25.4% of respondents considered free-roaming dogs to be a threat to their safety. When asked about the existence of free-roaming dogs, 95.1% of respondents stated that they preferred if there were no (77.1%) or fewer (18%) free-roaming dogs, and 74.8% disagreed with the presence of free-roaming dogs on the streets near their home or workplace. Finally, most respondents (76.4%) disagreed that dogs should be able to roam freely like wild animals and 77.4% disagreed that the benefits of free roaming outweigh the associated risks.

### 3.3. Attitudes Towards Free-Roaming Cats

Among 476 respondents, 83.4% reported having provided food to free-roaming cats, 78.6% reported providing water, 48.3% shelter, and 14.1% other care. Most respondents agreed that people should provide water, shelter, or food to free-roaming cats (Figure 3b). Respondents’ opinions on hygiene and public health risks varied, with 29.7% of respondents agreeing that free-roaming cats spread diseases and 24.8% agreeing that they spread litter and feces. Most of the respondents agreed that they were concerned about the welfare impacts of free roaming on the cats themselves (84.5%) or on companion cats (78.2%) but only 49.8% agreed with concerns about the impacts on wildlife and 22.7% reported that they have never seriously thought about it (12.6% neutral, 62% disagree). Most respondents (70.8%) agreed that predatory behavior of cats is a sign of normal behavior, but opinions varied regarding whether hunting other animals is necessary for cat welfare (34.2% disagreed, 26.9% neutral, 37.4% agreed). A small proportion (5.3%) of the respondents reported having felt physically threatened by a free-roaming cat, 5.9% reported having been attacked and 5.7% reported having been bitten or had seen a family member bitten by a free-roaming cat in the year preceding the survey. Most respondents (94.7%) disagreed with feeling physically threatened by free-roaming cats, even when it came to children (79.4%). When asked about the existence of free-roaming cats, 88.9% of respondents stated that they preferred if there were none (55.9%) or fewer (33%), and 59.5% of the respondents disagreed with the presence of free-roaming cats on the streets near their home or workplace. Finally, opinions varied on whether cats should roam freely like wild animals (59.7% disagree, 22.7% neutral, 15.5% agree), but over two thirds of the respondents (68.5%) disagreed that the benefits of cats roaming freely outweigh the risks.

**Figure 4:**
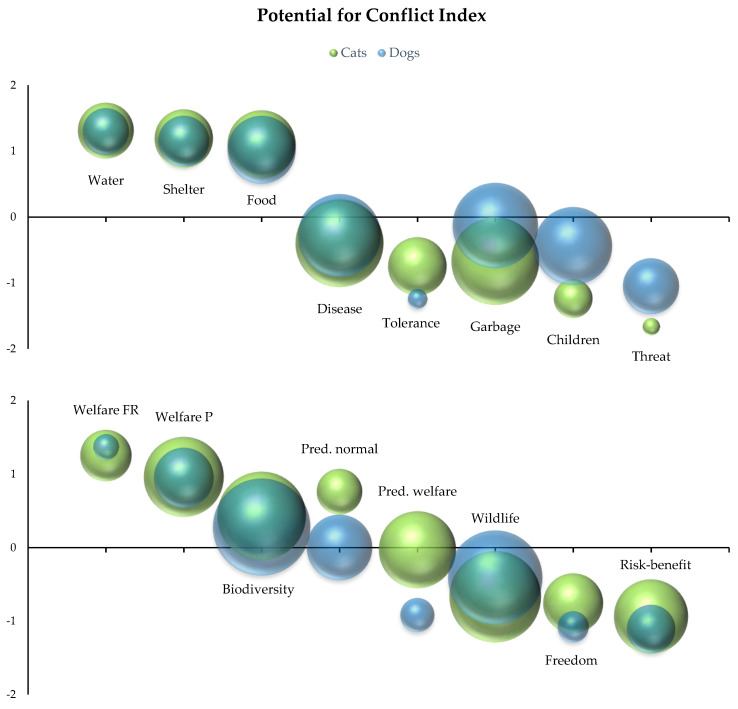
Potential conflict index scores for questions about attitudes toward free-roaming dogs and cats (−2: totally disagree, −1: disagree, 0: neither agree nor disagree, 1: agree, 2: totally agree). Legend: blue bubbles represent the answers of all respondents of the dog survey and green bubbles represent the answers of the cat survey. Labels represent the questions/statements as follows: water (“It is good that people provide water to free-roaming dogs/cats”), shelter (“It is good that people provide shelter to free-roaming dogs/cats”), food (“It is good that people feed free-roaming dogs/cats”), disease (“Free-roaming dogs/cats spread disease”), tolerance (“I like the presence of free-roaming dogs/cats around my home or work”), garbage (“Free-roaming dogs/cats spread garbage and feces”), children (“Free-roaming dogs/cats are a risk to the safety of children”), threat (“I feel physically threatened by free-roaming dogs/cats”), welfare FR (“I worry that free-roaming dogs/cats can be stolen, lost, or killed”), welfare P (“I worry that pet dogs or cats can be harmed by free-roaming dogs/cats”), biodiversity (“I am concerned about the impact of pet dogs/cats on biodiversity”), pred. normal (“If a dog/cat captures an animal, it is a sign of normal behavior”), pred. welfare (“Hunting is important for the welfare of dogs/cats”), wildlife (“I have never seriously thought about the impacts of dogs/cats on wildlife”), freedom (“Dogs/cats should be able to roam free, like wild animals”), and risk-benefit (“The benefits of free roaming outweigh the risks of being harmed or lost for dogs/cats”).

Data visualization using the potential for conflict index revealed differences in the dispersion (consensus) of Likert scale answers on attitudes on free-roaming animals in this survey, as well as differences between attitudes towards free-roaming cats and dogs (Figure 4). Answers regarding the provision of care (water, shelter, and food) to free-roaming animals had low dispersion and were similar for cats and dogs, favoring agreement. Answers regarding the potential spread of disease and garbage had high dispersion, and where similar between species in the case of disease, but not of garbage, where answers for dogs were centered near neutrality, and cats towards disagreement. Answers regarding the tolerance of free-roaming animals showed low dispersion for cats, and very low dispersion for dogs, both centered in the disagreement range. Regarding the threat that free-roaming animals pose to people and children, answers were centered in the disagreement range for both species, with very low dispersion for cats and low to moderate dispersion for dogs. Answers focusing on welfare concerns were centered in the agreement range for both species, with low to moderate dispersion for cats and very low to low dispersion for dogs. Answers related to impacts on biodiversity showed high dispersion for both species and were centered between neutrality and moderate agreement, with a similar trend to the statement that respondents had never seriously thought about the impacts on wildlife (formulated in the inverse sense). Regarding predatory behavior, answers regarding whether it signals normal behavior were centered around neutrality, with low dispersion for dogs, and centered in the range of agreement, with low dispersion for cats. Differences between species were also observed regarding the necessity of predatory behavior for their welfare, with answers for dogs centered in the disagreement range with very low dispersion and answers for cats centered around neutrality with moderate dispersion. Finally, answers on whether cats and dogs should be allowed to roam freely and whether the risks of free roaming outweigh the benefits were centered in the disagreement range for both species, with lower dispersion for dogs than for cats.

### 3.4. Factors That Influence Attitudes Toward Free-Roaming Animals

#### 3.4.1. “What Would You Prefer?”

**Figure 5:**
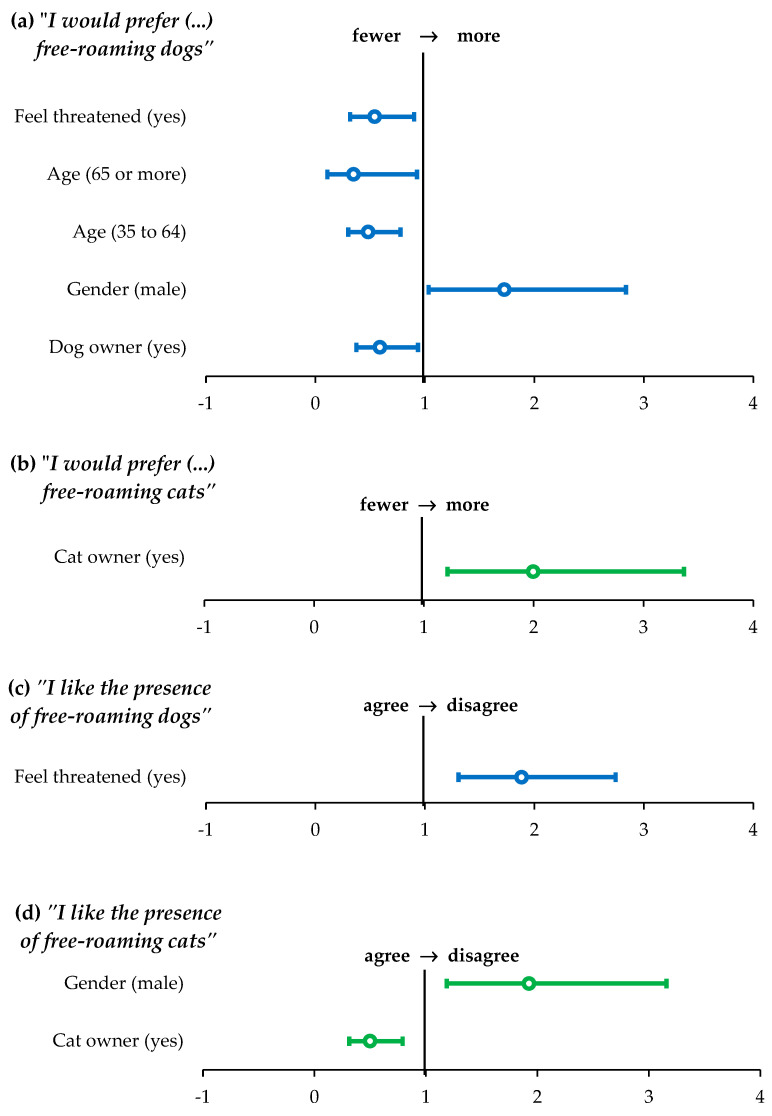
Factors that influence answers related to tolerance of free-roaming animals. Odds ratios (dots) and 95% confidence intervals (whisker bars) of the final models for the variation in the answers to the question “What would you prefer?” (no free-roaming dogs/cats, fewer free-roaming dogs/cat, I don’t mind, more free-roaming dogs/cats) (**a**,**b**); and agreement with the statement “I like the presence of free-roaming dogs/cats around my house or workplace” (**c**,**d**). Results are presented for dog (blue, *n* = 436) and cat (green, *n* = 373) owners.

The ordinal regression model for the question “What would you prefer?” (regarding the number of free-roaming dogs), allowed the calculation of the odds of respondents answering one level higher on the response scale of the ordinal variable (zero < fewer < don’t mind < more, free-roaming dogs/cats). For dogs, the factors retained in the final model were the gender and age of the respondent, whether they own dogs, and whether they feel physically threatened by free-roaming dogs (Figure 5). Respondents who owned dogs were associated with 61% lower odds (OR = 0.59; *p* = 0.024; 95% CI = 0.37–0.94) of responding one level higher on the scale, indicating a preference for fewer free-roaming dogs. Male respondents were associated with 1.73 times higher odds (OR = 1.73; *p* = 0.033; 95% CI = 1.03–2.84) of moving up one level on the scale, indicating greater tolerance for the existence of free-roaming dogs compared to female respondents. Finally, respondents aged 35 to 64 demonstrated 52% lower odds of moving up one level on the scale (OR = 0.48; *p* = 0.003; 95% CI = 0.30–0.78), as did those aged over 65 (65% lower odds, OR = 0.35; *p* = 0.049; 95% CI = 0.11–0.93), with both age groups showing less tolerance for the presence of free-roaming dogs compared to the 18 to 34 age group. Respondents who reported feeling physically threatened by free-roaming dogs were also associated with lower odds than others of responding one level higher on the scale (OR = 0.54; *p* = 0.021; 95% CI = 0.32–0.90), reflecting less tolerance for the existence of free-roaming dogs. In the case of cats (Figure 5), the only factor retained in the model for the same question was cat ownership. Respondents who report owning cats had twice the odds (OR = 2.00; *p* = 0.008; 95% CI = 1.21–3.37) of moving up one level on the response scale, indicating greater tolerance for the existence of free-roaming cats.

#### 3.4.2. “I Like the Presence of Free-Roaming Dogs/Cats Around My House or Workplace”

The ordinal model for this statement allows an estimation to be made of the odds (Figure 5) of respondents answering one level higher on the Likert scale (1 “strongly agree”, 2 “agree”, 3 “neither agree nor disagree”, 4 “disagree”, 5 “strongly disagree”). In the case of dogs (Figure 5), the only factor retained after model selection was the response to the question “I feel physically threatened by free-roaming dogs”. Respondents who reported feeling physically threatened by free-roaming dogs had odds 1.88 times higher of responding one level higher on the response scale (OR = 1.88; *p* = 0.001; 95% CI = 1.31–2.74), reflecting lower tolerance for the presence of free-roaming dogs near their home or workplace. In the case of cats, the retained factors were whether the gender of the respondent and whether he/she owned cats. Cat owners had 50% lower odds of responding one level higher on the scale (OR = 0.50; *p* = 0.003; 95% CI = 0.31–0.79), demonstrating greater tolerance towards free-roaming cats, while male respondents had odds 1.92 times higher of responding one level higher on the scale (OR = 1.92; *p* = 0.009; 95% CI = 1.19–3.16), reflecting lower tolerance towards free-roaming cats compared to female respondents.

### 3.5. Management of Free-Roaming Animals

**Figure 6:**
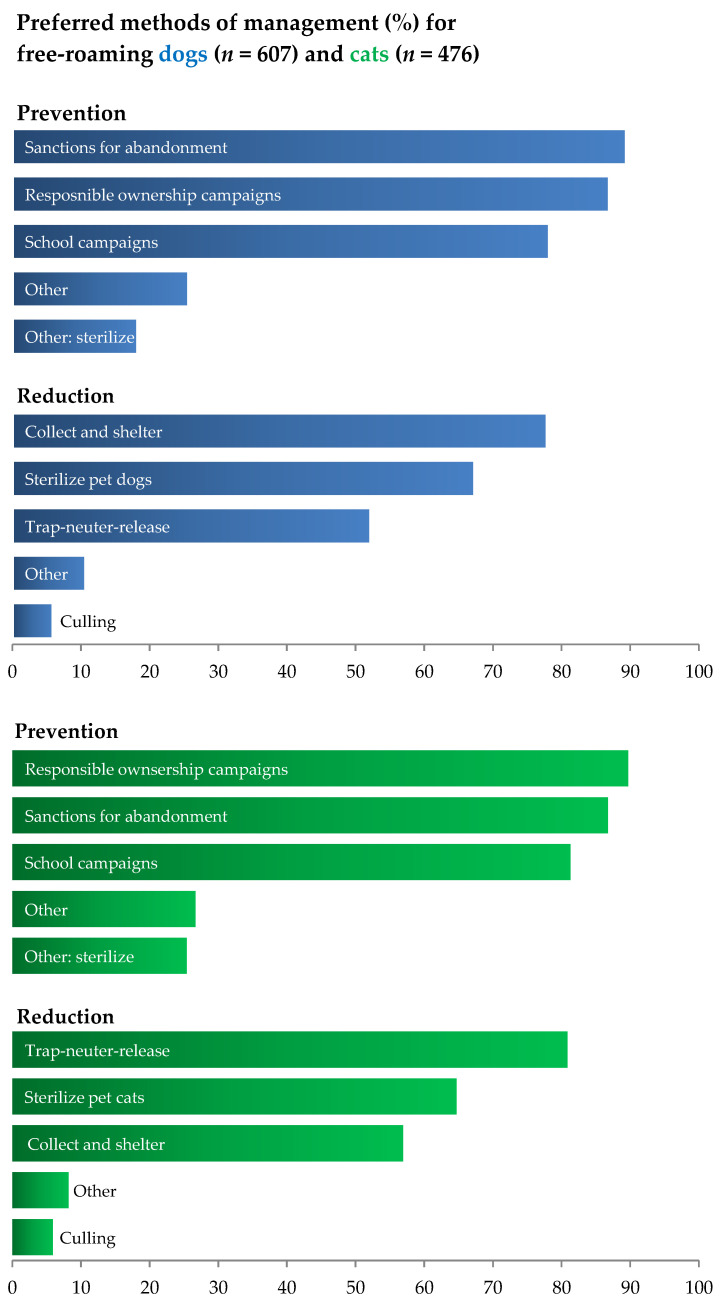
Preferred methods of management for free-roaming dogs and cats. Relative frequencies of non-mutually exclusive answers to the survey questions on the preferred ways to prevent and manage free-roaming dog (blue) and cat (green) populations.

When asked about ways to prevent the increase in the number of free-roaming dogs (Figure 6), 89.5% of respondents advocated for sanctions for animal abandonment, while others selected public campaigns for responsible ownership (87%) and educational campaigns in schools (78.3%). Regarding methods of reducing populations of free-roaming dogs, the option most frequently chosen was collection and sheltering (77.9%), followed by controlling the reproduction of owned dogs (67.4%) and trap–neuter–release programs (52.2%). In the case of free-roaming cats, 89.7% of respondents advocated for public campaigns for responsible ownership, as well as sanctions for animal abandonment (88.6%) and educational campaigns in schools (81.3%). Regarding methods of controlling populations of free-roaming cats, the option most frequently chosen was trap–neuter–release (80.9%), followed by controlling the reproduction of owned cats (64.7%) and collection and referral to shelters (56.9%).

Culling was advocated for by 5.9% of respondents for both dogs and cats, and in both cases, the responsibility for the management of free-roaming animals was predominantly attributed to government services (municipalities, state veterinarians, and the government). When questioned about the whether they had ever contacted anyone to report a free-roaming animal, the barriers most frequently reported by respondents were the fear that the animal would be culled (42% for dogs, 38% for cats), the fear that the animal would spend the rest of its life in a cage (30% for dogs, 34% for cats), and not knowing who to contact (27% for dogs, 24% for cats). Finally, when questioned about the main barriers to neutering pets, 46.3% and 50.1% of the dog and cat owners, respectively, refer to cost as the main barrier, while all other concerns (behavioral changes, health risks, weight gain, age, the right to breed, or religious objection) were chosen by less than 5% of the respondents.

## 4. Discussion

Strategies to address free-roaming populations of cats and dogs and to prevent and mitigate their impact need to be tailored and take social attitudes and perceptions into account. Our study identifies specific trends in a sample of adult online survey respondents residing in Portugal that can either thwart management efforts or be harnessed to potentiate them. Among them are specific areas for improvement in pet ownership practices or where additional effort is less likely to be efficient, specific attitudes and perceptions that may drive peoples’ decisions on providing resources or reporting a free-roaming animal, and preferred management practices or barriers.

### 4.1. Ownership Practices

Different patterns of pet ownership practices regarding cats and dogs were identified. Among practices that directly influence the dynamics of free-roaming animal populations or their impacts, allowing unrestricted or unsupervised access to the outdoor environment (29%) and the lack of identification and registration (35%) were identified as areas in need of improvement for cat owners. Considering the potential for social desirability bias and sampling bias towards welfare-engaged individuals with internet access, these results could be underestimated. The high frequency of cat sterilization (95%) is above the critical neutering fractions estimated, even for unrestricted free-roaming cats in colonies [56]. This suggests that pet cats are unlikely to function as a source for free-roaming cat populations. However, free-roaming sterilized pet cats are still capable of impacting biodiversity [57,58,59] and the time spent outside seems to be a determinant of predation [60,61]. Additionally, sterilized pet cats can have public health impacts and are vulnerable to the welfare issues associated with free roaming, such as collisions with vehicles, poisoning and disease. Therefore, a decrease in the unsupervised access of pet cats to the outdoor environment should be encouraged, as well as the increase in identification and registration, which can reduce the time to retrieval and welfare impacts in case of loss or theft [62,63,64]. Reducing unsupervised access to the outdoor environment does not necessarily require the continuous confinement of pet cats and can include middle-ground solutions such as outdoor enclosures, cat-proof fencing, and supervised access. Furthermore, confinement recommendations should consider contextual differences to ensure both effectiveness and social acceptability. Sterilization practices were more prevalent in our sample compared to those reported previously. For example, the reported sterilization rate of 95% in our sample was higher than the overall rate observed in India [65], Italy, Bulgaria, and Ukraine [5]. Reports of rabies vaccination were more prevalent in our sample compared to India [65], but similar to Italy, Bulgaria, and Ukraine [5]. Nevertheless, for dog owners in our study, the coverage of contraception or sterilization of the pet dog population is a potential area for improvement, since 20% of the owners in the survey report not using any form of contraception. This result should be interpreted considering the low percentage of owners who allow their dogs to roam unsupervised and unrestricted (8%), since low sterilization rates will only contribute to free-roaming dog populations in cases where these pet dogs are able to breed or are abandoned. Sterilization can have negative welfare impacts in dogs [66], which warrants a case-by-case harm–benefit analysis that can adequately balance stray dog population management objectives with the welfare of individual pet dogs. In situations where pet dogs are not allowed to roam, contraception of pet dogs might not contribute sufficiently to free-roaming population management to outweigh the welfare impacts. Responsible ownership practices of both cats and dogs in this sample were influenced by the area of residence, with rural owners presenting less odds of having a higher responsible ownership index. Geographic and demographic heterogeneity in ownership practices has also been identified in other regions [5,65], which reinforces the need for different approaches when considering management practices in rural or urban communities. Further, definitions of responsible ownership can vary based on cultural and geographic contexts and should be considered when designing effective and socially acceptable management interventions.

### 4.2. Attitudes

Our results revealed a consensual inclination towards the provision of food, water, and shelter to free-roaming cats and dogs. While potentially reflecting perspectives of individuals engaged in pet welfare issues due to sampling methods, if feeding is uncontrolled, it has the potential to contribute to the perpetuation of free-roaming populations. Unmanaged feeding can limit the success of trap–neuter–release programs by increasing the carrying capacity of the environment where these animals live [67,68]. Managed feeding, on the other hand, can provide opportunities for interventions that benefit the health and welfare of the animals themselves, people, and ecosystems. However, these interventions can be thwarted if uncontrolled feeding opportunities are present. Nevertheless, from the perspective of human–animal relations, this inclination to provide resources is often rooted in welfare concerns, kindness, and compassion [69]. A consensual concern for the welfare of free-roaming cats and dogs, and that the risks of roaming freely outweigh the benefits for them was also observed in our results, further supporting this relation. Altogether, these findings suggest that although the inclination to provide resources to free-roaming animals can limit the effectiveness of management programs, it is rooted in the same motivations that underly them. Therefore, redefining the concept of “helping free-roaming animals” to focus on the long-term welfare goal of reducing their populations, rather than the short-term goal of feeding them, is essential to align social attitudes with current management options.

Conversely, perceptions on the impacts on wildlife and public health were the least consensual and differed between cats and dogs. Respondents were consensual in agreeing that predation is not necessary for dog welfare, but a similar consensus was not observed for cats. Predatory behavior is commonly seen as a normal, natural, and even positive part of cat behavior [43]. However, when questioned about the need of this behavior to ensure welfare, the consensus decreased, with only 37% of respondents agreeing. Finally, only 25% of the respondents disagreed on having concerns for cats impacts on biodiversity, despite a high dispersion in the distribution of the answers (low consensus). Prior research has shown social attitudes towards cat predation to be dependent on culture and context [61], with low rates of concern with the impact of cats on biodiversity in some European countries [43,60,70]. In our case, the results suggest a scenario where, while considered a normal or natural behavior of cats, predation is not consensually considered necessary for cat welfare, and concern exists regarding the impacts of cats on biodiversity.

When the level of physical threat was considered, there was a consensual agreement that cats do not constitute a physical threat, and a similar agreement, although less consensual, was found for dogs. However, when the subjects at risk are children, the average score for the answers regarding dogs was close to the central point of the scale (neither agree nor disagree), with 25% of the respondents considering dogs a potential threat to children. These results highlight two different sets of perceptions and attitudes toward dogs and cats, which should be considered in tailored management approaches. The analysis of the factors influencing social tolerance for free-roaming dogs and cats also revealed species-specific scenarios. Older respondents and those that felt threatened by free-roaming dogs were less tolerant of their presence, while these factors were not retained in the models for cats. While these findings provide insights into how attitudes vary within our sample, caution is needed when extrapolating them to the general population, as older respondents are likely to be underrepresented due to the study’s online sampling approach. Being a cat owner increased the tolerance for free-roaming cats but being a dog owner decreased tolerance for free-roaming dogs. Similarly, male respondents were more tolerant to the presence of dogs, and less tolerant to the presence of cats. These results illustrate how social and demographic factors influence the attitudes toward free-roaming animals. While they are informative for Portuguese society at this point, caution is necessary when transposing them to the countries, as they are expected to vary temporally and geographically.

### 4.3. Management Practices

Sanctions for abandonment and responsible ownership campaigns were identified by more than 80% of the respondents as the preferred means to prevent the occurrence of free-roaming cats and dogs. While our self-selected sampling strategy may have led to overrepresentation of people concerned with animal welfare, broader data indicate that such concerns are widespread in Portugal. In a recent Eurobarometer survey [71], 87% of Portuguese respondents expressed concern for farm animal welfare and 89% for companion animal welfare, indicating that animal welfare is a broadly shared societal value. Hence, these results are likely to reflect the generalized concern for animal welfare in Portuguese people. Therefore, measures to increase the identification and registration of cats and to decrease unsupervised access to the outdoor environment could find widespread support if framed from an animal welfare perspective. Species-specific differences were observed in the preferred methods to reduce the populations of free-roaming animals. Collection and sheltering were the preferred method for dogs, followed by the sterilization of pet dogs. Cost was the main barrier to sterilization in our sample, which contrasts with findings in Bulgaria, Ukraine, and Italy [5] but is consistent with previous findings from Taiwan [72] and Brazil [73]. Furthermore, while religious beliefs and ownership for reasons other than companionship were negatively associated with dog neutering in other European countries [5], only a small proportion (0.7%) of the dog owners in our study referred to religion as a reason to avoid sterilization. The strong acceptance of the sterilization of pet dogs together with the referral of cost as the main barrier suggest that economic incentives for surgical sterilization are likely to be successful. However, these actions need to be considered in light of the low frequency of unsupervised outdoor access in the case of dogs and the already high proportion of sterilization in pet cats, which may render them redundant when applied to owned animals. The preference for trap–neuter–release interventions to reduce and control of free-roaming cat populations aligns with strong concern for cat welfare and their ability to express their natural behavior in our sample. These findings align with those from other studies (e.g., [41,74,75]), where trap–neuter–release strategies find most support among respondents, based on concerns for animal welfare. However, welfare concerns in our study were not accompanied by generalized concerns with impacts on biodiversity. These results align with previous trends where concerns with cat impacts are higher in areas where endemic biodiversity is high, such as in Australia and New Zealand, than in European countries [61,76,77]. Studies have documented various ecological consequences of free-roaming cats, including predation on native species, competition, and disease transmission [78]. However, the extent of these effects may vary with contexts, and studies tend to focus mostly on predation, which risks underestimating impacts on individual animals or non-lethal effects such as competition or fear (reviewed in [78]). The discussion on how to manage free-roaming dogs, and especially cats, has caused polarization between those with an ecological view and those with an animal welfare or animal rights view. This conflict arises from the difficulty of balancing the welfare of individual cats or dogs with the welfare of individual wild animals, and the value of ecosystems and their functions. However, we suggest that reconciling the views of an animal welfare-oriented people with those with ecological views is possible by redefining the social concept of what it means to ethically manage or help free-roaming animals. This can be done through educational campaigns focused on shifting public perception from the short-term goal of helping free-roaming animals by providing immediate and often short-lived resources or positive experiences to longer-term goals. Long-term goals can be described as reducing the number of free-roaming animals that are continuously vulnerable to traffic accidents, poisoning, hunger, thirst, harsh weather, persecution, and abandonment. Emphasizing these long-term objectives can help bridge the gap between ecological and animal welfare perspectives, enabling society to make strategic decisions on resource allocation and the means to feed, collect, rehabilitate, and rehome these animals. As with biodiversity impacts, our study did not find widespread concern with public health impacts of free-roaming dogs and cats. Results from previous studies are mixed, with some studies finding high levels of concern with disease transmission to humans, pets, and wildlife (e.g., [5,76]) and others finding limited concern with public health issues (e.g., [74]). Culling free-roaming animals was not seen as an acceptable practice by most of the respondents. The opposition to the use of lethal management methods is aligned with recent research [5,41,74,75], with lethal management methods finding support in only some regions such as Australia (e.g., [76]). In our study, the fear of culling was identified as a major barrier to reporting free-roaming animals, alongside spending the rest of their lives in a cage and not knowing who to contact. It is worth noting that the fear of culling persists among respondents despite its prohibition in Portugal by Law n.º 27/2016, enforced in September 2018, which could suggest distrust in institutions. These results indicate a need to rebuild confidence in the institutions tasked with the management of free-roaming animals, which should strive to increase their transparency and communication with the public. Failing to do so could limit the effectiveness of management plans, by reducing social compliance in reporting animals, or adherence plans to reduce resource provision. Finally, our study identified a strong support for sanctions on abandonment, educational campaigns to prevent the increase of free-roaming dog and cat populations. Our results are in line with prior research that has identified measures focusing on reducing pet abandonment and educational campaigns as valuable strategies [5,41,75], and suggest widespread support for them in Portugal.

### 4.4. Limitations

While the questionnaire was developed based on existing literature and expert input, it was not formally validated through psychometric testing, and potential measurement biases cannot be ruled out. Future studies should build on this foundation to conduct formal validation studies and enhance reliability and comparability. Additionally, the study relied on self-reported data collected through an online survey, which introduces self-selection bias and limits generalizability. The sample may overrepresent individuals with strong interest in pet ownership and animal welfare, as well as those with access to digital platforms. According to recent data, 86–88% of Portuguese adults use the internet regularly [79]. Nevertheless, bias due to access to digital platforms can occur (e.g., due to lower access among older individuals and those in rural areas). Finally, the external validity of the findings should be interpreted with caution. While this study provides valuable insights into social attitudes towards free-roaming animals, it is based on convenience sampling and may not fully capture the diversity of the general population. Future research could refine these findings by employing probability-based sampling methods or combining online surveys with alternative strategies to improve representativeness.

## 5. Conclusions

This study provides a snapshot of the perceptions and attitudes that influence free-roaming animal populations in Portugal and highlights the shortfalls and opportunities. Key improvements in pet ownership include the need to increase cat identification and registration, to reduce unsupervised outdoor access for pet cats, and to encourage the sterilization of pet dogs. Regarding attitudes, our findings indicate a strong welfare-driven perspective, with widespread support for providing resources such as food, water, and shelter to free-roaming animals. In terms of free-roaming animal management, the study identified strong support for responsible ownership campaigns and sanctions on abandonment, limited support for lethal management approaches, and species-specific preferences, with trap–neuter–release supported for cats and collection and sheltering preferred for dogs. These findings suggest that management strategies emphasizing animal welfare concerns may align more closely with public attitudes. The balance between ecological and welfare perspectives remains challenging. While concern for the welfare of free-roaming animals was high, perceptions of their environmental impact were more divided, particularly for cats.

While our study relies on a convenience sample of internet users, online surveys provide a valuable means to reach a broad audience and capture societal attitudes. Future research using complementary methodologies could further refine these insights. Despite these limitations, our findings offer a relevant basis for the development of socially informed policies.

## Figures and Tables

**Table 1:** Distribution of absolute (count) and relative frequencies (proportion) of responsible ownership index scores for cat and dog owners among survey respondents.

	Dogs		Cats	
Responsible Ownership Index Score	Count	Proportion (%)	Count	Proportion (%)
0	1	0.23%	4	1.07%
1	0	0.00%	8	2.14%
2	3	0.69%	18	4.83%
3	8	1.83%	42	11.26%
4	37	8.49%	68	18.23%
5	125	28.67%	122	32.71%
6	262	60.09%	111	29.76%
Total (*n*)	436		373	

**Table 2:** List of independent variables used to fit candidate models. Variables retained in final models are highlighted in bold.

**Model 1:**	**Responsible Ownership Index**					
**Species**	**Species (Dogs)**			**Species (Cats)**		
	**Df**	**LRT**	***p*-Value**	**Df**	**LRT**	***p*-Value**
Motivation for ownership	**1**	**4.45**	0.03 *	**1**	**6.62**	0.01 *
Respondent adopts pets	**1**	**6.36**	0.01 *	1	0.7	0.4
Respondent acquires pets	1	0.47	0.49	1	3.62	0.06
Number of owned animals	2	3.94	0.14	2	1.26	0.53
Respondent age	2	1.94	0.38	2	4.22	0.12
Respondent gender	1	3.2	0.07	1	0.34	0.56
Marital status	4	6.37	0.17	**4**	**11.46**	0.02 *
Respondent education	2	1.66	0.44	2	2.32	0.31
Occupation	6	8.12	0.23	6	2.81	0.83
Number of residents in the home	4	9.21	0.06	4	7.63	0.11
Number of children in the home	1	2.37	0.12	1	0.22	0.64
Type of area of residence	**2**	**8.66**	0.01 *	**2**	**11.95**	<0.01 *
**Model 2:**	**“What would you prefer? (no free-roaming dogs/cats, fewer free-roaming dogs/cats, I don’t mind, more free-roaming dogs/cats)”**					
**Species**	**Species (Dogs)**			**Species (Cats)**		
	**Df**	**LRT**	***p*-value**	**Df**	**LRT**	***p*-value**
Owner	**1**	**4.85**	0.03 *	**1**	**5.84**	0.02 *
Respondent gender	**1**	**5.15**	0.02 *	1	0.25	0.62
Respondent age	**2**	**7.06**	0.03 *	2	0.73	0.69
Respondent education	2	1.99	0.37	2	0.51	0.77
Number of children in the home	1	0.71	0.4	1	0.03	0.87
Type of area of residence	2	3.04	0.22	2	1.89	0.39
Respondent feels threatened	**1**	**5.51**	0.02 *	1	0.69	0.41
Someone bitten in the last 12 mo.	1	0.01	0.91	1	3.18	0.07
**Model 3:**	**“I like the presence of free-roaming dogs/cats around my house or workplace (strongly agree, agree, neither agree nor disagree, disagree, strongly disagree)”**					
**Species**	**Species (Dogs)**			**Species (Cats)**		
	**Df**	**LRT**	***p*-value**	**Df**	**LRT**	***p*-value**
Owner	1	0.78	0.38	**1**	**7.95**	<0.01 *
Respondent gender	1	0.08	0.78	**1**	**7.74**	0.01 *
Respondent age	2	0.81	0.67	2	0.2	0.91
Respondent education	2	0.43	0.81	2	0.06	0.97
Number of children in the home	1	0.09	0.76	1	1.65	0.2
Type of area of residence	2	3.79	0.15	2	4.51	0.1
Respondent feels threatened	**1**	**11.87**	<0.01 *	1	0.79	0.37
Someone bitten in the last 12 mo.	1	1.44	0.23	1	1.59	0.21

Legend: the table presents results for model selection based on candidate ordinal regression models. LRT: Likelihood Ratio Test, testing whether removing a variable significantly reduces model fit. Df: Degrees of freedom, representing the number of independent parameters being tested. Significant *p*-values (*p* < 0.05) indicate variables retained in final models and are highlighted in bold, and with a * next to the *p*-value.

**Table 3:** Unadjusted and adjusted odds ratios (ORs) for factors associated with responsible ownership index scores in dog and cat owners.

Variable	Unadjusted OR (95% CI)	Adjusted OR (95% CI)	*p*-Value
**Dogs (*n* = 390)**			
Motivation: companionship	reference	reference	-
**Motivation: other**	**4.88 (NA–NA)**	**0.33 (0.17–0.63)**	**0.001**
Adoption: no	reference	reference	-
**Adoption: yes**	**1.81 (0.07–46.0)**	**2.07 (1.36–3.16)**	**0.001**
Type of area of residence: peri-urban	reference	reference	-
**Type of area of residence: rural/natural area**	**0.73 (0.03–18.7)**	**0.57 (0.34–0.96)**	**0.036**
Type of area of residence: urban	1.72 (NA–NA)	1.32 (0.82–2.15)	0.256
**Cats (*n* = 334)**			
Motivation: companionship	reference	reference	-
**Motivation: other**	**0.24 (0.09–0.72)**	**0.41 (0.20–0.87)**	**0.020**
Marital status: married	reference	reference	-
Marital status: cohabiting	0.17 (0.02–0.73)	0.66 (0.37–1.17)	0.157
Marital status: divorced	0.27 (0.03–1.70)	0.68 (0.35–1.34)	0.264
**Marital status: single**	**0.15 (0.02–0.56)**	**0.55 (0.34–0.90)**	**0.016**
Marital status: widowed	0.14 (0.01–3.29)	0.38 (0.11–1.27)	0.114
Type of area of residence: peri-urban	reference	reference	-
Type of area of residence: rural/natural area	0.66 (0.25–1.83)	0.81 (0.46–1.42)	0.452
**Type of area of residence: urban**	**1.78 (0.69–4.63)**	**2.13 (1.36–3.35)**	**0.001**

Legend: this table presents unadjusted odds ratios (ORs) with 95% confidence intervals (CIs) and adjusted odds ratios with 95% confidence intervals and *p*-values for variables associated with responsible ownership index scores in dog and cat owners. Bold indicates significant variables, with *p* < 0.05 and 95% confidence interval not overlapping with 1. Model sample sizes (*n* = 390 for dogs, *n* = 334 for cats) differ from total survey respondents due to exclusions of cases with missing data (na.exclude in R). NA values in CIs of unadjusted ORs occurred due to statistical instability caused by low counts in categories of some variables, leading to separation issues where the model cannot reliably estimate an effect size. In such cases, the adjusted model provides a more stable and interpretable estimate after controlling for confounders.

## Data Availability

The data used in this study are part of the 2023 Portuguese National Census of Free-Roaming Animals, managed under specific confidentiality conditions. As such, these data are not publicly accessible to ensure compliance with privacy and ethical considerations. However, the raw data supporting the conclusions of this article will be made available by the corresponding author upon reasonable request, provided such requests comply with ethical guidelines and the policies governing the Portuguese National Census of Free-Roaming Animals.

## Supplementary Materials

The following supporting information can be downloaded at: https://www.mdpi.com/article/10.3390/ani15060771/s1, File S1: final versions of the questionnaires; Table S1: social and demographic distribution of the respondents.
