# Supplementary material for: Social Perceptions and Attitudes Towards Free-Roaming Cats and Dogs in Portugal: An Exploratory Study
Source: Animals (Basel). 2025 Mar 8;15(6):771. doi: 10.3390/ani15060771 (PMC11939513; doi:10.3390/ani15060771)
Supplement: Supplementary file 1 [file animals-15-00771-s001.zip › File S1.pdf]

Social perceptions and attitudes towards free-roaming cats and dogs in Portugal

S1 File. Online questionnaires for dogs and cats.

Inquérito Cães Errantes v.ativa

Existe(m) 50 questão(ões) neste questionário.

Inquérito Cães Errantes

Convidamo-lo(a) a participar num estudo que aborda a perceção da sociedade portuguesa sobre a problemática dos cães errantes (a designação de cães errantes inclui por regra animais abandonados, animais nascidos sem detentor, mas também animais que têm detentor, mas que acabam por passar uma parte do dia em áreas de domínio público e/ou em áreas privadas que não as dos respetivos proprietários). Este estudo está enquadrado no projeto "Censo Nacional dos Animais Errantes 2023" levado a cabo pelo Instituto da Conservação da Natureza e das Florestas (https://www.icnf.pt/), Departamento de Biologia (https://www.ua.pt/pt/dbio) e CESAM - Centro de Estudos do Ambiente e do Mar (http://www.cesam.ua.pt/) da Universidade de Aveiro (https://www.ua.pt/) e financiado pelo Fundo Florestal Permanente.

O objetivo deste estudo é recolher informação sobre as perceções sociais em relação a cães e gatos errantes, bem como as formas mais adequadas de gerir as populações destes animais, salvaguardando o seu bem-estar. Prevê-se que o preenchimento do inquérito possa demorar entre 10 a 15 minutos e a sua participação é totalmente voluntária. Em caso de necessidade, pode gravar as suas respostas para submeter mais tarde (selecione "Continuar mais tarde"). O inquérito só termina após selecionar "Submeter". A sua participação neste estudo é confidencial, e garantimos a segurança da sua informação mantendo o anonimato em todas as fases do estudo, desde a recolha da informação nos inquéritos até à análise e publicação dos resultados. Todos os dados recolhidos são anónimos.

Para eventuais dúvidas contacte-nos através do email bio-animais.errantes@ua.pt

A. Confirmo que: (i) li e entendi a apresentação e explicação do estudo; (ii) compreendo o estudo e tomei conhecimento das vias de contacto disponíveis para esclarecer eventuais dúvidas; e (iii) tomei conhecimento da possibilidade de abandonar o inquérito antes de o submeter.

B. Concordo com o armazenamento e utilização dos dados que vou submeter para investigação relacionada com o tema, sempre de uma forma anónima.

*

Por favor, selecionar no máximo uma resposta
Por favor, selecione **todas** as que se aplicam:

- Sim
- Não

A sua participação neste inquérito terminou.

Responda a esta pergunta apenas se as seguintes condições são verdadeiras:
A resposta for 'Não' na pergunta '2 [Q00001]' (A. Confirmo que: (i) li e entendi a apresentação e explicação do estudo; (ii) compreendo o estudo e tomei conhecimento das vias de contacto disponíveis para esclarecer eventuais dúvidas; e (iii) tomei conhecimento da possibilidade de abandonar o inquérito antes de o submeter. B. Concordo com o armazenamento e utilização dos dados que vou submeter para investigação relacionada com o tema, sempre de uma forma anónima.)

Secção 1) Práticas de manutenção ou detenção de cães

Como mantemos os nossos cães de estimação

Atualmente é detentor (dono/tutor/guardião) de pelo menos um cão?

Por favor, selecione **apenas uma** das seguintes opções:

- Sim
- Não
- Não respondo

Quantos cães tem?

Responda a esta pergunta apenas se as seguintes condições são verdadeiras:
A resposta for 'Sim' na pergunta '4 [Q00002]' (Atualmente é detentor (dono/tutor/guardião) de pelo menos um cão?)
Por favor, selecione a posição apropriada para cada elemento:

	0	1	2	3	4	5	Mais de 5	Não respondo
**Machos**								
**Fêmeas**								

Indique o número de cães que possui por cada uma das seguintes classes de idade:

Responda a esta pergunta apenas se as seguintes condições são verdadeiras:
A resposta for 'Sim' na pergunta '4 [Q00002]' (Atualmente é detentor (dono/tutor/guardião) de pelo menos um cão?)
Por favor, selecione a posição apropriada para cada elemento:

	0	1	2	3	4	5	6	7	8	9	Mais de 10	Não respondo
**Menos de 1 ano**												
**1 a 3 anos**												
**Mais de 3 anos**												

Os seus cães estão identificados com microchip e registados no SIAC?

Responda a esta pergunta apenas se as seguintes condições são verdadeiras:
A resposta for 'Sim' na pergunta '4 [Q00002]' (Atualmente é detentor (dono/tutor/guardião) de pelo menos um cão?)
Por favor, selecione **apenas uma** das seguintes opções:

- Sim, todos
- Sim, alguns
- Não, nenhum
- Não sei
- Não respondo

Qual o principal motivo pelo qual tem cães? Por favor selecione o número de opções necessárias.

Responda a esta pergunta apenas se as seguintes condições são verdadeiras:
A resposta for 'Sim' na pergunta '4 [Q00002]' (Atualmente é detentor (dono/tutor/guardião) de pelo menos um cão?)
Por favor, selecione **todas** as que se aplicam:

- Por prazer e/ou companhia, isto é, como animal de estimação
- Por utilidade, por exemplo guarda ou caça
- Não respondo
- Outro (por favor indique qual):

Onde adquiriu os seus cães (assinale todas as opções necessárias)?

Responda a esta pergunta apenas se as seguintes condições são verdadeiras:
A resposta for 'Sim' na pergunta '4 [Q00002]' (Atualmente é detentor (dono/tutor/guardião) de pelo menos um cão?)

Por favor, selecione **todas** as que se aplicam:

- Abrigo de animais
- Internet
- Loja de animais
- Criador
- Criação em minha casa
- Encontrado
- De um amigo ou familiar
- Não respondo
- Outro (por favor indique qual):

Pagou pelo(s) seu(s) cão(cães)?

Responda a esta pergunta apenas se as seguintes condições são verdadeiras:
A resposta for 'Sim' na pergunta '4 [Q00002]' (Atualmente é detentor (dono/tutor/guardião) de pelo menos um cão?)
Por favor, selecione **apenas uma** das seguintes opções:

- Sim, por todos
- Sim, por alguns
- Não
- Não respondo

Que idade tinha(m) o(s) seu(s) cão(cães) quando veio (vieram) para casa?

Responda a esta pergunta apenas se as seguintes condições são verdadeiras:
A resposta for 'Sim' na pergunta '4 [Q00002]' (Atualmente é detentor (dono/tutor/guardião) de pelo menos um cão?)
Por favor, selecione **apenas uma** das seguintes opções:

- Todos cãezinhos
- Cãezinhos e adultos
- Todos adultos
- Não respondo

Algum dos seus cães ou cadelas já teve crias?

Responda a esta pergunta apenas se as seguintes condições são verdadeiras:
A resposta for 'Sim' na pergunta '4 [Q00002]' (Atualmente é detentor (dono/tutor/guardião) de pelo menos um cão?)
Por favor, selecione **apenas uma** das seguintes opções:

- Sim
- Não
- Não sei
- Não respondo

Considerando todos os seus cães, quantas vezes no total tiveram crias?

Responda a esta pergunta apenas se as seguintes condições são verdadeiras:
A resposta for 'Sim' na pergunta '4 [Q00002]' (Atualmente é detentor (dono/tutor/guardião) de pelo menos um cão?) *e* A resposta for 'Sim' na pergunta '12 [Q00011]' (Algum dos seus cães ou cadelas já teve crias?)
Por favor, selecione **apenas uma** das seguintes opções:

- Uma vez
- Duas vezes
- Três ou mais vezes
- Não respondo

O que fez com os cãezinhos? Por favor indique todas as opções que se apliquem.

Responda a esta pergunta apenas se as seguintes condições são verdadeiras:
A resposta for 'Sim' na pergunta '4 [Q00002]' (Atualmente é detentor (dono/tutor/guardião) de pelo menos um cão?) *e* A resposta for 'Sim' na pergunta '12 [Q00011]' (Algum dos seus cães ou cadelas já teve crias?)
Por favor, selecione **todas** as que se aplicam:

- Fiquei com eles
- Entreguei os cãezinhos a um abrigo
- Telefonei às autoridades
- Dei os cãezinhos a amigo(s)/conhecido(s)
- Vendi os cãezinhos
- Deixei os cãezinhos livres na rua
- Levei os cãezinhos para eutanasiar numa cliníca
- Não respondo
- Outro (por favor indique qual):

Como faz para impedir/prevenir a reprodução (ou criação) dos seus cães?

Responda a esta pergunta apenas se as seguintes condições são verdadeiras:
A resposta for 'Sim' na pergunta '4 [Q00002]' (Atualmente é detentor (dono/tutor/guardião) de pelo menos um cão?)
Por favor, selecione **todas** as que se aplicam:

- Esterilização cirúrgica (castração ou ovariohisterectomia)
- Impedindo o contacto entre machos e fêmeas
- Pílula/injeção
- Não impeço/previno
- Não respondo
- Outro (por favor indique qual):

Na sua opinião, quais serão os principais motivos para não impedir que os cães criem ou reproduzam?

Responda a esta pergunta apenas se as seguintes condições são verdadeiras:
A resposta for 'Sim' na pergunta '4 [Q00002]' (Atualmente é detentor (dono/tutor/guardião) de pelo menos um cão?)
Por favor, selecione **todas** as que se aplicam:

- As esterilizações ou castrações são caras
- Um cão ou cadela deve reproduzir (criar) pelo menos uma vez
- Acho que o meu animal de estimação é novo de mais para ser esterilizado
- A esterilização/castração é incompatível com as minhas crenças religiosas
- A esterilização/castração provoca aumento de peso
- A esterilização/castração provoca alterações no comportamento
- A esterilização/castração é um risco para a saúde do animal
- Os cães têm o direito de se reproduzir livremente
- Nenhum, não há motivo para não controlar a reprodução
- Não respondo
- Outro (por favor indique qual):

Responda a esta pergunta apenas se as seguintes condições são verdadeiras:
A resposta for 'Sim' na pergunta '4 [Q00002]' (Atualmente é detentor (dono/tutor/guardião) de pelo menos um cão?)
Por favor, selecione a posição apropriada para cada elemento:

	**Sim**	**Não**	**Não respondo**
**Alimenta os seus cães todos os dias?**			
**Dá água aos seus cães todos os dias?**			
**Providencia abrigo aos seus cães todos os dias?**			
**Vacina os seus cães contra a raiva?**			
**Vacina os seus cães contra outras doenças?**			

Permite que algum dos seus cães tenha acesso à rua, circulando para fora da sua propriedade sem estar acompanhado?

Responda a esta pergunta apenas se as seguintes condições são verdadeiras:
A resposta for 'Sim' na pergunta '4 [Q00002]' (Atualmente é detentor (dono/tutor/guardião) de pelo menos um cão?)
Por favor, selecione **apenas uma** das seguintes opções:

- Sempre
- Às vezes
- Nunca
- Não respondo

Indique em que período(s) do dia o(s) seu(s) cão(cães) costuma(m) ficar mais tempo fora de casa sem supervisão.

Responda a esta pergunta apenas se as seguintes condições são verdadeiras:
A resposta for 'Sim' na pergunta '4 [Q00002]' (Atualmente é detentor (dono/tutor/guardião) de pelo menos um cão?) *e* A resposta for 'Sempre' *ou* 'Às vezes' na pergunta '18 [Q00018]' (Permite que algum dos seus cães tenha acesso à rua, circulando para fora da sua propriedade sem estar acompanhado?)
Por favor, selecione **todas** as que se aplicam:

- Manhã
- Tarde
- Noite
- Amanhecer e/ou entardecer (crepúsculo)
- Todo o dia
- Não respondo

Quanto tempo é que o(s) seu(s) cão(cães) costuma(m) ficar fora de casa sem supervisão?

Responda a esta pergunta apenas se as seguintes condições são verdadeiras:
A resposta for 'Sim' na pergunta '4 [Q00002]' (Atualmente é detentor (dono/tutor/guardião) de pelo menos um cão?) *e* A resposta for 'Sempre' *ou* 'Às vezes' na pergunta '18 [Q00018]' (Permite que algum dos seus cães tenha acesso à rua, circulando para fora da sua propriedade sem estar acompanhado?)
Por favor, selecione **apenas uma** das seguintes opções:

- Menos de 1 hora
- Entre 1 a 4 horas
- Entre 4 a 8 horas
- Mais de 8 horas
- Não respondo

Algum dos seus cães alguma vez caçou um animal?

Responda a esta pergunta apenas se as seguintes condições são verdadeiras:
A resposta for 'Sim' na pergunta '4 [Q00002]' (Atualmente é detentor (dono/tutor/guardião) de pelo menos um cão?)
Por favor, selecione **apenas uma** das seguintes opções:

- Sim
- Não
- Não respondo

Assinale todas as presas que o(s) seu(s) cão(cães) já caçou(caçaram).

Responda a esta pergunta apenas se as seguintes condições são verdadeiras:
A resposta for 'Sim' na pergunta '4 [Q00002]' (Atualmente é detentor (dono/tutor/guardião) de pelo menos um cão?) *e* A resposta for 'Sim' na pergunta '21 [Q00021]' (Algum dos seus cães alguma vez caçou um animal?)
Por favor, selecione **todas** as que se aplicam:

- Rato
- Lagartixa
- Inseto

- Ave
- Coelho
- Não respondo
- Outro (por favor indique qual):

Alguma vez teve que desistir, dar ou abdicar de um cão?

Responda a esta pergunta apenas se as seguintes condições são verdadeiras:
A resposta for 'Sim' na pergunta '4 [Q00002]' (Atualmente é detentor (dono/tutor/guardião) de pelo menos um cão?)
Por favor, selecione **apenas uma** das seguintes opções:

- Sim
- Não
- Não respondo

Como o fez?

Responda a esta pergunta apenas se as seguintes condições são verdadeiras:
A resposta for 'Sim' na pergunta '4 [Q00002]' (Atualmente é detentor (dono/tutor/guardião) de pelo menos um cão?) *e* A resposta for 'Sim' na pergunta '23 [Q00023]' (Alguma vez teve que desistir, dar ou abdicar de um cão?)
Por favor, selecione **todas** as que se aplicam:

- Entreguei a um abrigo
- Telefonei às autoridades
- Dei a amigo(s)/conhecido(s)
- Vendi
- Libertei
- Eutanásia numa clínica
- Não respondo
- Outro (por favor indique qual):

Qual foi o motivo?

Responda a esta pergunta apenas se as seguintes condições são verdadeiras:
A resposta for 'Sim' na pergunta '4 [Q00002]' (Atualmente é detentor (dono/tutor/guardião) de pelo menos um cão?) *e* A resposta for 'Sim' na pergunta '23 [Q00023]' (Alguma vez teve que desistir, dar ou abdicar de um cão?)
Por favor, selecione **apenas uma** das seguintes opções:

- Perdi o interesse
- Um problema de comportamento animal
- Motivos económicos
- Não respondo
- Outro (por favor indique qual)

Secção 2) Atitudes em relação aos cães errantes

A designação de **cães errantes** inclui por regra animais abandonados, animais nascidos sem detentor, mas também animais que têm detentor, mas que acabam por passar uma parte do dia em áreas de domínio público e/ou em áreas privadas que não as dos respetivos proprietários.

Quando foi a última vez que viu um cão errante?

Por favor, selecione **apenas uma** das seguintes opções:

- Hoje
- Na última semana
- No último mês
- No último ano

- Nunca
- Não respondo

Por favor, selecione a posição apropriada para cada elemento:

	Sim	Não	Não respondo
**Alguma vez se sentiu fisicamente ameaçado(a) por um cão errante?**			
**Alguma vez foi atacado(a) por um cão errante?**			
**Nos últimos 12 meses, foi mordido(a) (ou alguém da sua família) por um cão errante?**			

Alguma vez providenciou cuidados a cães errantes? Escolha todas as opções necessárias.

Por favor, selecione **todas** as que se aplicam:

- Alimento
- Água
- Abrigo
- Nenhum
- Não respondo
- Outro (por favor indique qual):

Por favor indique se concorda ou discorda das seguintes afirmações, conforme a escala de concordância:

Por favor, selecione a posição apropriada para cada elemento:

	Discordo totalmente	Discordo	Não concordo nem discordo	Concordo	Concordo totalmente	Não respondo
**Gosto que existam cães errantes nas ruas perto da minha habitação ou local de trabalho.**						
**É bom que as pessoas providenciem abrigos para os cães errantes.**						
**É bom que as pessoas alimentem os cães errantes.**						
**É bom que as pessoas dêem água aos cães errantes.**						
**Sinto-me fisicamente ameaçado(a) por cães errantes.**						
**Os cães errantes espalham doenças.**						
**Os cães errantes são uma ameaça para a segurança das crianças.**						
**Os cães errantes espalham lixo e fezes.**						

Por favor indique se concorda ou discorda das seguintes afirmações, conforme a escala de concordância:

Por favor, selecione a posição apropriada para cada elemento:

	Discordo totalmente	Discordo	Não concordo nem discordo	Concordo	Concordo totalmente	Não respondo
**Preocupa-me que os cães de estimação possam ser magoados por outros**						

	Discordo totalmente	Discordo	Não concordo nem discordo	Concordo	Concordo totalmente	Não respondo
**animais quando andam soltos (outros cães, animais selvagens, etc.).**						
**Preocupo-me com a possibilidade de cães errantes serem roubados, perdidos ou mortos.**						
**Preocupo-me com o impacto dos cães de estimação sobre a vida selvagem.**						
**Nunca pensei seriamente sobre os impactos dos cães na vida selvagem.**						
**Os cães devem poder andar livres, como os animais silvestres.**						
**Os benefícios dos cães andarem soltos são maiores do que os riscos de se magoarem ou perderem.**						
**Se um cão caçar um animal, é sinal de que tem um comportamento normal.**						
**Caçar é importante para o bem-estar de um cão.**						

Na sua opinião, quem deveria ser responsável por gerir os cães errantes, como por exemplo providenciar-lhes cuidados e prevenir o aumento do seu número? Escolha os três mais importantes.

Por favor, selecionar no máximo 3 respostas
Por favor, selecione **todas** as que se aplicam:

- O Governo
- As Câmaras Municipais
- Veterinários do Estado
- Veterinários privados
- Forças policiais
- Organizações de voluntários
- Serviços de gestão de resíduos
- Ninguém
- Não respondo
- Outro (por favor indique qual):

Como acha que se pode prevenir o aumento de cães errantes? Escolha todas as opções que considerar necessárias.

Por favor, selecione **todas** as que se aplicam:

- Campanhas públicas de sensibilização para a detenção responsável
- Campanhas de educação nas escolas
- Sanções para quem abandone animais
- Não acho que se deva prevenir/evitar o aumento do número de cães errantes
- Não respondo
- Outro (por favor indique qual):

O que preferia:

Por favor, selecione a posição apropriada para cada elemento:

Que não existissem cães errantes	Que existissem menos cães errantes	Não me importo que existam cães errantes	Que existissem mais cães errantes	Não respondo

Como acha que deve ser reduzido o número de cães errantes? Escolha todas as opções necessárias.

Por favor, selecione **todas** as que se aplicam:

- Recolhendo os cães e levando-os para abrigos
- Capturar e esterilizar os cães, devolvendo-os depois à rua
- Controlando o ritmo de reprodução dos cães com detentor (dono/tutor)
- Através de eutanásia
- Não acho que se deva reduzir o número de cães errantes
- Não respondo
- Outro (por favor indique qual):

Alguma vez contactou alguém (ou alguma instituição) para que um cão errante fosse recolhido?

Por favor, selecione **apenas uma** das seguintes opções:

- Sim
- Não
- Não respondo

Assinale qual/quais o(s) motivo(s).

Responda a esta pergunta apenas se as seguintes condições são verdadeiras:
A resposta for 'Não' na pergunta '35 [Q00037]' (Alguma vez contactou alguém (ou alguma instituição) para que um cão errante fosse recolhido?   )
Por favor, selecione **todas** as que se aplicam:

- Nunca vi nenhum cão nesta situação
- Na minha opinião, os cães errantes não são um problema
- Tenho receio que os cães recolhidos sejam abatidos
- Não quero que os cães passem o resto da vida numa jaula
- Não sabia quem contactar
- Não respondo
- Outro (por favor indique qual):

Indique a pessoa ou instituição que contactou:

Responda a esta pergunta apenas se as seguintes condições são verdadeiras:

A resposta for 'Sim' na pergunta '35 [Q00037]' (Alguma vez contactou alguém (ou alguma instituição) para que um cão errante fosse recolhido?   )
Por favor, selecione **todas** as que se aplicam:

- Um veterinário municipal ou um centro de recolha oficial
- Uma associação que ajuda animais
- A polícia
- Um veterinário privado
- Não respondo
- Outro (por favor indique qual):

Secção 3) Informação sociodemográfica

Qual é o seu país de residência?

Por favor, selecione **apenas uma** das seguintes opções:

- Portugal
- Outro (por favor indique qual)

Indique os primeiros quatro números do seu código postal (por exemplo 3810):

Neste campo só é possível introduzir números.
Por favor, escreva aqui a sua resposta:

Em que distrito reside?

Por favor, selecione **apenas uma** das seguintes opções:

- Não respondo
- Aveiro
- Beja
- Braga
- Bragança
- Castelo Branco
- Coimbra
- Évora
- Faro
- Guarda
- Leiria
- Lisboa
- Portalegre
- Porto
- Santarém
- Setúbal
- Viana do Castelo
- Vila Real
- Viseu

Qual é a sua nacionalidade?

Por favor, selecione **apenas uma** das seguintes opções:

- Portuguesa
- Outra (por favor indique qual)

Qual das seguintes opções melhor descreve a sua habitação?

Por favor, selecione **apenas uma** das seguintes opções:

- Apartamento ou moradia sem zona exterior (jardim, pátio, varanda)
- Apartamento ou moradia com zona exterior vedada (jardim, pátio, varanda, terreno)
- Apartamento ou moradia com zona exterior **não** vedada (jardim, pátio, varanda, terrenos agrícolas, etc.)

Qual das seguintes opções melhor descreve a sua área de residência?

Por favor, selecione **apenas uma** das seguintes opções:

- Área urbana
- Área periurbana
- Área rural/natural

Idade:

Por favor, selecione **apenas uma** das seguintes opções:

- Menos de 18
- 18 a 24
- 25 a 34
- 35 a 44
- 45 a 54
- 55 a 64
- 65 a 74
- 75 ou mais
- Não respondo

Qual o seu género?

Por favor, selecione **apenas uma** das seguintes opções:

- Masculino
- Feminino
- Não respondo
- Outro (por favor indique)

Qual a sua ocupação principal?

Por favor, selecione **apenas uma** das seguintes opções:

- Trabalhador/empresário no ativo
- Desempregado
- Estudante
- Em busca de emprego
- Em casa, tarefas domésticas
- Reformado
- Não respondo
- Outro (por favor indique qual)

Qual o seu nível de escolaridade?

Por favor, selecione **apenas uma** das seguintes opções:

- Sem nível de escolaridade
- Ensino Básico (entre as idades de 5 e 12 anos)
- Ensino Secundário (entre as idades de 11 e 18 anos)
- Ensino Superior (Universidade ou politécnicos)
- Não respondo

Religião:

Por favor, selecione **apenas uma** das seguintes opções:

- Não respondo
- Sem religião
- Budista
- Católica
- Cristã Ortodoxa
- Hindu
- Judaica
- Muçulmana
- Protestante
- Outra (por favor indique qual)

Estado civil:

Por favor, selecione **apenas uma** das seguintes opções:

- Solteiro(a)
- Casado(a)
- Em coabitação (ou união de facto)
- Divorciado(a)
- Viúvo(a)
- Não respondo

Por favor, selecione a posição apropriada para cada elemento:

	0	1	2	3	4	5	Mais de 5	Não respondo
**Número de pessoas que habitam na sua residência:**								
**Número de crianças (menores de 16 anos) que habitam na sua residência:**								

Agradecemos a participação neste inquérito relativo ao Censo Nacional de Animais Errantes 2021/2022.
05.05.2024 – 15:32

Submeter o seu inquérito
Obrigado por ter concluído este inquérito.

Inquérito Gatos Errantes v.ativa

Existe(m) 50 questão(ões) neste questionário.

Inquérito Gatos Errantes

Convidamo-lo(a) a participar num estudo que aborda a perceção da sociedade portuguesa sobre a problemática dos gatos errantes (a designação de gatos errantes inclui por regra animais abandonados, animais nascidos sem detentor, mas também animais que têm detentor, mas que acabam por passar uma parte do dia em áreas de domínio público e/ou em áreas privadas que não as dos respetivos proprietários). Este estudo está enquadrado no projeto "Censo Nacional dos Animais Errantes 2023" levado a cabo pelo Instituto da Conservação da Natureza e das Florestas (https://www.icnf.pt/), Departamento de Biologia (https://www.ua.pt/pt/dbio) e CESAM - Centro de Estudos do Ambiente e do Mar (http://www.cesam.ua.pt/) da Universidade de Aveiro (https://www.ua.pt/) e financiado pelo Fundo Florestal Permanente.

O objetivo deste estudo é recolher informação sobre as perceções sociais em relação a cães e gatos errantes, bem como as formas mais adequadas de gerir as populações destes animais, salvaguardando o seu bem-estar. Prevê-se que o preenchimento do inquérito possa demorar entre 10 a 15 minutos e a sua participação é totalmente voluntária. Em caso de necessidade, pode gravar as suas respostas para submeter mais tarde (selecione "Continuar mais tarde"). O inquérito só termina após selecionar "Submeter". A sua participação neste estudo é confidencial, e garantimos a segurança da sua informação mantendo o anonimato em todas as fases do estudo, desde a recolha da informação nos inquéritos até à análise e publicação dos resultados. Todos os dados recolhidos são anónimos.

Para eventuais dúvidas contacte-nos através do email bio-animais.errantes@ua.pt

A. Confirmo que: (i) li e entendi a apresentação e explicação do estudo; (ii) compreendo o estudo e tomei conhecimento das vias de contacto disponíveis para esclarecer eventuais dúvidas; e (iii) tomei conhecimento da possibilidade de abandonar o inquérito antes de o submeter.

B. Concordo com o armazenamento e utilização dos dados que vou submeter para investigação relacionada com o tema, sempre de uma forma anónima.

*

Por favor, selecione **todas** as que se aplicam:

- Sim
- Não

A sua participação neste inquérito terminou.

Secção 1) Práticas de manutenção ou detenção de gatos

Como mantemos os nossos gatos de estimação

Atualmente é detentor (dono/tutor/guardião) de pelo menos um gato?

Por favor, selecione **apenas uma** das seguintes opções:

- Sim
- Não
- Não respondo

Quantos gatos tem?

Por favor, selecione a posição apropriada para cada elemento:

	0	1	2	3	4	5	Mais de 5	Não respondo
**Machos**								
**Fêmeas**								

Indique o número de gatos que possui por cada uma das seguintes classes de idade:

Por favor, selecione a posição apropriada para cada elemento:

	0	1	2	3	4	5	6	7	8	9	Mais de 10	Não respondo
**Menos de 1 ano**												
**1 a 3 anos**												
**Mais de 3 anos**												

Os seus gatos estão identificados com microchip e registados no SIAC?

Por favor, selecione **apenas uma** das seguintes opções:

- Sim, todos
- Sim, alguns
- Não, nenhum
- Não sei
- Não respondo

Qual o principal motivo pelo qual tem gatos? Por favor selecione o número de opções necessárias.

Por favor, selecione **todas** as que se aplicam:

- Por prazer e/ou companhia, isto é, como animal de estimação
- Por utilidade, por exemplo controlo de roedores

- Não respondo
- Outro (por favor indique qual):

Onde adquiriu os seus gatos (assinale todas as opções necessárias)?

Por favor, selecione **todas** as que se aplicam:

- Abrigo de animais
- Internet
- Loja de animais
- Criador
- Criação em minha casa
- Encontrado
- De um amigo ou familiar
- Não respondo
- Outro (por favor indique qual):

Pagou pelo(s) seu(s) gato(s)?

Por favor, selecione **apenas uma** das seguintes opções:

- Sim, por todos
- Sim, por alguns
- Não
- Não respondo

Que idade tinha(m) o(s) seu(s) gato(s) quando veio (vieram) para casa?

Por favor, selecione **apenas uma** das seguintes opções:

- Todos gatinhos
- Gatinhos e adultos
- Todos adultos
- Não respondo

Algum dos seus gatos ou gatas já teve crias?

Por favor, selecione **apenas uma** das seguintes opções:

- Sim
- Não
- Não sei
- Não respondo

Considerando todos os seus gatos, quantas vezes no total tiveram crias?

Por favor, selecione **apenas uma** das seguintes opções:

- Uma vez
- Duas vezes
- Três ou mais vezes
- Não respondo

O que fez com os gatinhos? Por favor indique todas as opções que se apliquem.

Por favor, selecione **todas** as que se aplicam:

- Fiquei com eles

- Entreguei os gatinhos a um abrigo
- Telefonei às autoridades
- Dei os gatinhos a amigo(s)/conhecido(s)
- Vendi os gatinhos
- Deixei os gatinhos livres na rua
- Levei os gatinhos para eutanasiar numa cliníca
- Não respondo
- Outro (por favor indique qual):

Como faz para impedir/prevenir a reprodução (ou criação) dos seus gatos?

Por favor, selecione **todas** as que se aplicam:

- Esterilização cirúrgica (castração ou ovariohisterectomia)
- Impedindo o contacto entre machos e fêmeas
- Pílula/injeção
- Não impeço/previno
- Não respondo
- Outro (por favor indique qual):

Na sua opinião, quais serão os principais motivos para não impedir que os gatos criem ou reproduzam?

Por favor, selecione **todas** as que se aplicam:

- As esterilizações ou castrações são caras
- Um gato ou gata deve reproduzir (criar) pelo menos uma vez
- Acho que o meu animal de estimação é novo de mais para ser esterilizado
- A esterilização/castração é incompatível com as minhas crenças religiosas
- A esterilização/castração provoca aumento de peso
- A esterilização/castração provoca alterações no comportamento
- A esterilização/castração é um risco para a saúde do animal
- Os gatos têm o direito de se reproduzir livremente
- Nenhum, não há motivo para não controlar a reprodução
- Não respondo
- Outro (por favor indique qual):

Por favor, selecione a posição apropriada para cada elemento:

	Sim	Não	Não respondo
**Alimenta os seus gatos todos os dias?**			
**Dá água aos seus gatos todos os dias?**			
**Providencia abrigo aos seus gatos todos os dias?**			
**Vacina os seus gatos?**			
**Vacina os seus gatos contra a FeLV (leucemia felina)?**			

Permite que algum dos seus gatos tenha acesso à rua, circulando para fora da sua propriedade sem estar acompanhado?

Por favor, selecione **apenas uma** das seguintes opções:

- Sempre
- Às vezes
- Nunca
- Não respondo

Indique em que período(s) do dia o(s) seu(s) gato(s) costuma(m) ficar mais tempo fora de casa sem supervisão.

Por favor, selecione **todas** as que se aplicam:

- Manhã
- Tarde
- Noite
- Amanhecer e/ou entardecer (crepúsculo)
- Todo o dia
- Não respondo

Quanto tempo é que o(s) seu(s) gato(s) costuma(m) ficar fora de casa sem supervisão?

Por favor, selecione **apenas uma** das seguintes opções:

- Menos de 1 hora
- Entre 1 a 4 horas
- Entre 4 a 8 horas
- Mais de 8 horas
- Não respondo

Algum dos seus gatos alguma vez trouxe um "presente" para casa (caçou um animal)?

Por favor, selecione **apenas uma** das seguintes opções:

- Sim
- Não
- Não respondo

Assinale todas as presas que o(s) seu(s) gato(s) já caçou(caçaram).

Por favor, selecione **todas** as que se aplicam:

- Rato
- Lagartixa
- Inseto
- Ave
- Coelho
- Não respondo
- Outro (por favor indique qual):

Alguma vez teve que desistir, dar ou abdicar de um gato?

Por favor, selecione **apenas uma** das seguintes opções:

- Sim
- Não
- Não respondo

Como o fez?

Por favor, selecione **todas** as que se aplicam:

- Entreguei a um abrigo
- Telefonei às autoridades
- Dei a amigo(s)/conhecido(s)
- Vendi
- Libertei
- Eutanásia numa clínica
- Não respondo
- Outro (por favor indique qual):

Qual foi o motivo?

Por favor, selecione **apenas uma** das seguintes opções:

- Perdi o interesse
- Um problema de comportamento animal
- Motivos económicos
- Não respondo
- Outro (por favor indique qual)

Secção 2) Atitudes em relação aos gatos errantes

A designação de **gatos errantes** inclui por regra animais abandonados, animais nascidos sem detentor, mas também animais que têm detentor, mas que acabam por passar uma parte do dia em áreas de domínio público e/ou em áreas privadas que não as dos respetivos proprietários.

Quando foi a última vez que viu um gato errante?

Por favor, selecione **apenas uma** das seguintes opções:

- Hoje
- Na última semana
- No último mês
- No último ano
- Nunca
- Não respondo

Por favor, selecione a posição apropriada para cada elemento:

	Sim	Não	Não respondo
**Alguma vez se sentiu fisicamente ameaçado(a) por um gato errante?**			
**Alguma vez foi atacado(a) por um gato errante?**			
**Nos últimos 12 meses, foi mordido(a)/arranhado(a) (ou alguém da sua família) por um gato errante?**			

Alguma vez providenciou cuidados a gatos errantes? Escolha todas as opções necessárias.

Por favor, selecione **todas** as que se aplicam:

- Alimento
- Água
- Abrigo
- Nenhum
- Não respondo
- Outro (por favor indique qual):

Por favor indique se concorda ou discorda das seguintes afirmações, conforme a escala de concordância:

Por favor, selecione a posição apropriada para cada elemento:

	Discordo totalmente	Discordo	Não concordo nem discordo	Concordo	Concordo totalmente	Não respondo
**Gosto que existam gatos errantes nas ruas perto da minha habitação ou local de trabalho.**						

	Discordo totalmente	Discordo	Não concordo nem discordo	Concordo	Concordo totalmente	Não respondo
**É bom que as pessoas providenciem abrigos para os gatos errantes.**						
**É bom que as pessoas alimentem os gatos errantes.**						
**É bom que as pessoas dêem água aos gatos errantes.**						
**Sinto-me fisicamente ameaçado(a) por gatos errantes.**						
**Os gatos errantes espalham doenças.**						
**Os gatos errantes são uma ameaça para a segurança das crianças.**						
**Os gatos errantes espalham lixo e fezes.**						

Por favor indique se concorda ou discorda das seguintes afirmações, conforme a escala de concordância:

Por favor, selecione a posição apropriada para cada elemento:

	Discordo totalmente	Discordo	Não concordo nem discordo	Concordo	Concordo totalmente	Não respondo
**Preocupa-me que os gatos de estimação possam ser magoados por outros animais quando andam soltos (outros cães, animais selvagens, etc.).**						
**Preocupo-me com a possibilidade de gatos errantes serem roubados, perdidos ou mortos.**						
**Preocupo-me com o impacto dos gatos de estimação sobre a vida selvagem.**						
**Nunca pensei seriamente sobre os impactos dos gatos na vida selvagem.**						
**Os gatos devem poder andar livres, como os animais silvestres.**						
**Os benefícios dos gatos andarem soltos são maiores do que os riscos de se magoarem ou perderem.**						
**Se um gato caçar um animal, é sinal de que tem um comportamento normal.**						
**Caçar é importante para o bem-estar de um gato.**						

Na sua opinião, quem deveria ser responsável por gerir os gatos errantes, como por exemplo providenciar-lhes cuidados e prevenir o aumento do seu número? Escolha os três mais importantes.

Por favor, selecione **todas** as que se aplicam:

- O Governo
- As Câmaras Municipais
- Veterinários do Estado
- Veterinários privados
- Forças policiais

- Organizações de voluntários
- Serviços de gestão de resíduos
- Ninguém
- Não respondo
- Outro (por favor indique qual):

Como acha que se pode prevenir o aumento de gatos errantes? Escolha todas as opções que considerar necessárias.

Por favor, selecione **todas** as que se aplicam:

- Campanhas públicas de sensibilização para a detenção responsável
- Campanhas de educação nas escolas
- Sanções para quem abandone animais
- Não acho que se deva prevenir/evitar o aumento do número de gatos errantes
- Não respondo
- Outro (por favor indique qual):

O que preferia:

Por favor, selecione a posição apropriada para cada elemento:

Que não existissem gatos errantes	Que existissem menos gatos errantes	Não me importo que existam gatos errantes	Que existissem mais gatos errantes	Não respondo

Como acha que deve ser reduzido o número de gatos errantes? Escolha todas as opções necessárias.

Por favor, selecione **todas** as que se aplicam:

- Recolhendo os gatos e levando-os para abrigos
- Capturar e esterilizar os gatos, devolvendo-os depois à rua
- Controlando o ritmo de reprodução dos gatos com detentor (dono/tutor)
- Através de eutanásia
- Não acho que se deva reduzir o número de gatos errantes
- Não respondo
- Outro (por favor indique qual):

Alguma vez contactou alguém (ou alguma instituição) para que um gato errante fosse recolhido?

Por favor, selecione **apenas uma** das seguintes opções:

- Sim
- Não
- Não respondo

Assinale qual/quais o(s) motivo(s).

Por favor, selecione **todas** as que se aplicam:

- Nunca vi nenhum gato nesta situação
- Na minha opinião, os gatos errantes não são um problema
- Tenho receio que os gatos recolhidos sejam abatidos
- Não quero que os gatos passem o resto da vida numa jaula
- Não sabia quem contactar
- Não respondo

- Outro (por favor indique qual):

Indique a pessoa ou instituição que contactou:

Por favor, selecione **todas** as que se aplicam:

- Um veterinário municipal ou um centro de recolha oficial
- Uma associação que ajuda animais
- A polícia
- Um veterinário privado
- Não respondo
- Outro (por favor indique qual):

Secção 3) Informação sociodemográfica

Qual é o seu país de residência?

Por favor, selecione **apenas uma** das seguintes opções:

- Portugal
- Outro (por favor indique qual)

Indique os primeiros quatro números do seu código postal (por exemplo 3810):

Por favor, escreva aqui a sua resposta:

Em que distrito reside?

Por favor, selecione **apenas uma** das seguintes opções:

- Não respondo
- Aveiro
- Beja
- Braga
- Bragança
- Castelo Branco
- Coimbra
- Évora
- Faro
- Guarda
- Leiria
- Lisboa
- Portalegre
- Porto
- Santarém
- Setúbal
- Viana do Castelo
- Vila Real
- Viseu

Qual é a sua nacionalidade?

Por favor, selecione **apenas uma** das seguintes opções:

- Portuguesa
- Outra (por favor indique qual)

Qual das seguintes opções melhor descreve a sua habitação?

Por favor, selecione **apenas uma** das seguintes opções:

- Apartamento ou moradia sem zona exterior (jardim, pátio, varanda)
- Apartamento ou moradia com zona exterior vedada (jardim, pátio, varanda, terreno)
- Apartamento ou moradia com zona exterior **não** vedada (jardim, pátio, varanda, terrenos agrícolas, etc.)

Qual das seguintes opções melhor descreve a sua área de residência?

Por favor, selecione **apenas uma** das seguintes opções:

- Área urbana
- Área periurbana
- Área rural/natural

Idade:

Por favor, selecione **apenas uma** das seguintes opções:

- Menos de 18
- 18 a 24
- 25 a 34
- 35 a 44
- 45 a 54
- 55 a 64
- 65 a 74
- 75 ou mais
- Não respondo

Qual o seu género?

Por favor, selecione **apenas uma** das seguintes opções:

- Masculino
- Feminino
- Não respondo
- Outro (por favor indique)

Qual a sua ocupação principal?

Por favor, selecione **apenas uma** das seguintes opções:

- Trabalhador/empresário no ativo
- Desempregado
- Estudante
- Em busca de emprego
- Em casa, tarefas domésticas
- Reformado
- Não respondo
- Outro (por favor indique qual)

Qual o seu nível de escolaridade?

Por favor, selecione **apenas uma** das seguintes opções:

- Sem nível de escolaridade
- Ensino Básico (entre as idades de 5 e 12 anos)

- Ensino Secundário (entre as idades de 11 e 18 anos)
- Ensino Superior (Universidade ou politécnicos)
- Não respondo

Religião:

Por favor, selecione **apenas uma** das seguintes opções:

- Não respondo
- Sem religião
- Budista
- Católica
- Cristã Ortodoxa
- Hindu
- Judaica
- Muçulmana
- Protestante
- Outra (por favor indique qual)

Estado civil:

Por favor, selecione **apenas uma** das seguintes opções:

- Solteiro(a)
- Casado(a)
- Em coabitação (ou união de facto)
- Divorciado(a)
- Viúvo(a)
- Não respondo

Por favor, selecione a posição apropriada para cada elemento:

	0	1	2	3	4	5	Mais de 5	Não respondo
**Número de pessoas que habitam na sua residência:**								
**Número de crianças (menores de 16 anos) que habitam na sua residência:**								

Agradecemos a participação neste inquérito relativo ao Censo Nacional de Animais Errantes 2021/2022.
05.05.2024 – 15:31

Submeter o seu inquérito
Obrigado por ter concluído este inquérito.
