# Supplementary material for: Social Perceptions and Attitudes Towards Free-Roaming Cats and Dogs in Portugal: An Exploratory Study
Source: Animals (Basel). 2025 Mar 8;15(6):771. doi: 10.3390/ani15060771 (PMC11939513; doi:10.3390/ani15060771)
Supplement: Supplementary file 1 [file animals-15-00771-s001.zip › Table S1.pdf]

Social perceptions and attitudes towards free-roaming cats and dogs in Portugal

S2 Table. Social and demographic distribution of the respondents. Social and demographic distribution of the respondents to the questionnaires on perceptions and attitudes towards free-roaming dogs and cats.

Questionnaire – dogs

**Country of residence**	Portugal	607	99%
	Other*	3	0.5%
	No answer*	4	0.7%
**Nationality**	Portuguese	594	98%
	Other	9	1.5%
	No answer	11	2%
**Age**	18_to_24	55	9%
	25_to_34	104	17%
	35_to_44	124	20%
	45_to_54	147	24%
	55_to_64	128	21%
	65_to_74	45	7%
	75_or_more	3	0.5%
	No answer	1	0.2%
**Gender**	Female	481	79%
	Male	111	18%
	Other	1	0.2%
	No answer	14	2%
**Occupation**	Active worker	422	70%
	Student	67	11%
	Retired	55	9%
	Unemployed	30	5%
	Household work	12	2%
	Searching for work	11	2%
	Other	4	0.7%
	No answer	6	1.0%
**Education**	University education	446	73%
	High school	138	23%
	Primary school	15	2%
	No answer	8	1.3%
**Civil state**	Single	210	35%
	Married	189	31%
	Cohabiting	99	16%
	Divorced	76	13%
	Widowed	16	3%
	No answer	17	3%
**Dog owner**	Yes	436	72%
	No	168	28%
	No answer	3	0.5%

**Number of people living in the household**	1	89	15%
	2	236	39%
	3	153	25%
	4	95	16%
	5	19	3%
	>5	1	0.2%
	No answer	14	2%
**Number of people under 16 living in the household**	0	473	78%
	1	73	12%
	2	22	4%
	3	5	0.8%
	4	0	0.0%
	5	0	0.0%
	>5	0	0.0%
	No answer	34	6%
**District**	Lisboa	141	23%
	Aveiro	128	21%
	Porto	63	10%
	Setubal	60	10%
	Leiria	32	5%
	Coimbra	31	5%
	Santarem	25	4%
	Beja	20	3%
	Faro	18	3%
	Castelo Branco	15	2%
	Braga	14	2%
	Évora	9	1.5%
	Guarda	9	1.5%
	Viana do Castelo	9	1.5%
	Vila Real	9	1.5%
	Viseu	9	1.5%
	Braganca	5	0.8%
	Portalegre	5	0.8%
	Açores	3	0.5%
	No answer	2	0.3%
**Average duration of survey**		00:12:55	

*excluded answers (n=7), only answers from people residing in Portugal were considered (n=607)

Questionnaire – cats

**Country of residence**	Portugal	476	98.6%
	Other*	0	0.6%
	No answer*	7	0.8%
**Nationality**	Portuguese	457	96.0%
	Other	11	2.3%
	No answer	8	1.7%
**Age**	18_to_24	51	10.7%
	25_to_34	84	17.6%
	35_to_44	98	20.6%
	45_to_54	118	24.8%
	55_to_64	83	17.4%
	65_to_74	34	7.1%
	75_or_more	6	1.3%
	No answer	2	0.4%
**Gender**	Female	387	81.3%
	Male	81	17.0%
	Other	1	0.2%
	No answer	7	1.5%
**Occupation**	Active worker	312	65.3%
	Student	64	13.4%
	Retired	42	8.8%
	Unemployed	31	6.5%
	Household work	10	2.1%
	Searching for work	9	1.9%
	Other	3	0.6%
	No answer	5	1.0%
**Education**	University education	366	76.9%
	High school	97	20.4%
	Primary school	12	2.5%
	No answer	3	0.6%
**Civil state**	Single	171	35.9%
	Married	142	29.8%
	Cohabiting	82	17.2%
	Divorced	55	11.6%
	Widowed	9	1.9%
	No answer	17	3.6%
**Cat owner**	Yes	373	78.4%
	No	101	21.2%
	No answer	2	0.4%

**Number of people living in the household**	1	75	16%
	2	187	39%
	3	117	25%
	4	69	14%
	5	14	3%
	>5	1	0.2%
	No answer	13	3%
**Number of people under 16 living in the household**	0	384	81%
	1	53	11%
	2	12	3%
	3	4	0.8%
	4	0	0.0%
	5	1	0.2%
	>5	0	0.0%
	No answer	22	5%
**District**	Lisboa	115	19%
	Aveiro	106	17%
	Porto	59	10%
	Setúbal	37	6%
	Coimbra	24	4%
	Leiria	21	3%
	Santarém	21	3%
	Beja	18	3%
	Castelo Branco	18	3%
	Braga	12	2%
	Viana do Castelo	8	1%
	Viseu	8	1.3%
	Vila Real	7	1.2%
	Évora	6	1.0%
	Faro	5	0.8%
	Portalegre	4	0.7%
	Guarda	3	0.5%
	Açores	2	0.3%
	No answer	2	0.3%
**Average duration of survey**	00:11:00		

*excluded answers (n=7), only answers from people residing in Portugal were considered (n=476)
